# sangeranalyseR: Simple and Interactive Processing of Sanger Sequencing Data in R

**DOI:** 10.1093/gbe/evab028

**Published:** 2021-02-16

**Authors:** Kuan-Hao Chao, Kirston Barton, Sarah Palmer, Robert Lanfear

**Affiliations:** 1 Department of Ecology and Evolution, Research School of Biology, Australian National University, Canberra, Australian Capital Territory, Australia; 2 The Garvan Institute of Medical Research, Darlinghurst, New South Wales, Australia; 3 The University of Sydney School of Medicine, New South Wales, Australia

**Keywords:** genetics, DNA, alignment, bioconductor, shiny application, chromatogram

## Abstract

sangeranalyseR is feature-rich, free, and open-source R package for processing Sanger sequencing data. It allows users to go from loading reads to saving aligned contigs in a few lines of R code by using sensible defaults for most actions. It also provides complete flexibility for determining how individual reads and contigs are processed, both at the command-line in R and via interactive Shiny applications. sangeranalyseR provides a wide range of options for all steps in Sanger processing pipelines including trimming reads, detecting secondary peaks, viewing chromatograms, detecting indels and stop codons, aligning contigs, estimating phylogenetic trees, and more. Input data can be in either ABIF or FASTA format. sangeranalyseR comes with extensive online documentation and outputs aligned and unaligned reads and contigs in FASTA format, along with detailed interactive HTML reports. sangeranalyseR supports the use of colorblind-friendly palettes for viewing alignments and chromatograms. It is released under an MIT licence and available for all platforms on Bioconductor (https://bioconductor.org/packages/sangeranalyseR, last accessed February 22, 2021) and on Github (https://github.com/roblanf/sangeranalyseR, last accessed February 22, 2021).

**Significance**

Sequencing technology has improved dramatically over the last decade, and new sequencing technologies have been accompanied by a plethora of excellent and interoperable bioinformatic tools. But bioinformatic tools for older sequencing methods have not kept pace. Sanger sequencing is one of the most widely used sequencing methods in the world. But the processing of Sanger sequencing often requires the use of cumbersome and/or expensive software that can be difficult to integrate with other packages. sangeranslyseR solves this problem—it is a free and open-source R/Bioconductor package that provides simple and flexible functions for all commonly performed tasks. It can be used to build fast and reproducible workflows, has extensive documentation, includes an intuitive GUI, and exposes the results of Sanger sequencing experiments to the huge range of available analysis tools in R and Bioconductor. We hope that sangeranalyseR will improve and democratize the analysis of Sanger sequencing data.

## Introduction

Sanger sequencing (Sanger and Coulson 1975; Sanger et al. 1977) was the first controllable method to determine nucleic acid sequences and was commercialized by Applied Biosystems in 1986. Although it has been more than forty years since it was first proposed in 1977, and many new sequencing methods have since been introduced, it is still widely used and indispensable for sequencing individual DNA fragments and validating the results of Next-generation sequencing projects (Kircher and Kelso 2010; Stucky 2012). There are many widely used tools for processing Sanger sequencing reads, such as Geneious (Kearse et al. 2012), CodonCode Aligner (CodonCode Corporation, Dedham, MA), Phred-Phrap-Consed (Ewing and Green 1998), and Sequencher (Gene Codes Corporation, Ann Arbor, MI). However, these tools tend to be either expensive or have restrictive licences that limit their use, and there are no such tools built into the popular R ecosystem for bioinformatic analyses. As a result, the analysis of Sanger sequencing data is often more expensive and/or more difficult than the analysis of data from more recently developed sequencing platforms.

The R language is increasingly popular for bioinformatic analyses, and is thus an attractive language for which to develop a new package for the analysis of Sanger sequencing data. One R package, sangerseqR (Hill et al. 2014), focuses on the analysis of individual Sanger sequencing reads. But no current R packages are dedicated to solving the challenges of assembling multiple reads into contigs. To address this, we present sangeranalyseR: An automated R package for the processing of Sanger sequencing data. sangeranalyseR builds extensively on the excellent sangerseqR package but is focused on constructing multiple contigs from multiple Sanger sequencing reads. It provides: quality trimming, base calling, chromatogram plotting, assembly of contigs from any number of forward and reverse reads, contig alignment, phylogenetic tree reconstruction, and a number of additional methods to analyze reads and contigs in more detail. The package includes two interactive local Shiny applications which allow users to look in detail into each read and contig and change input parameters, such as those used for read trimming. sangeranalyseR is available on Bioconductor, is free and open source, and includes extensive documentation hosted by ReadTheDocs at https://sangeranalyser.readthedocs.io/en/latest/ (last accessed February 22, 2021).

## Overview

**Fig. 1.**
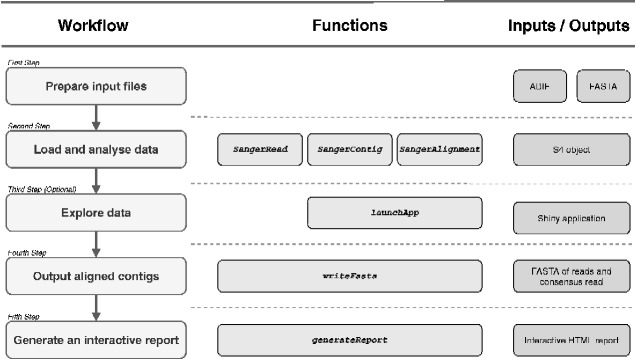
The five step sangeranalyseR analysis workflow. (Column 1) The steps of a simple analysis using sangeranalyseR. The third step, exploring data through Shiny applications, is optional. (Column 2) The corresponding functions invoked in each step of the analysis in column 1. After preparing input files, users can run *SangerRead*, *SangerContig*, or *SangerAlignment* to start an analysis. The different functions produce objects containing a single read, a single contig, or a collection of aligned (and so assumed alignable contigs). Shiny applications are only available for the *SangerContig* and *SangerAlignment* objects. (Column 3) The input and/or output files that correspond to each step of the analysis.

**Fig. 2.**
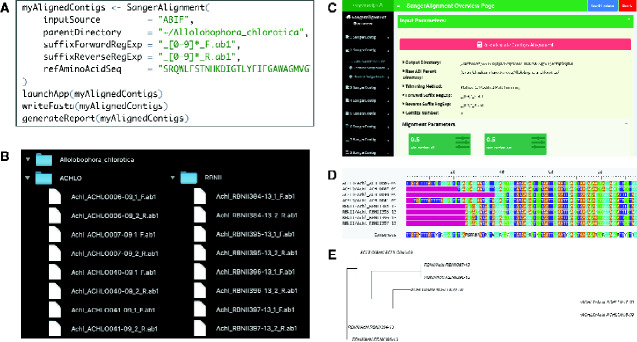
An example of creating a *SangerAlignment* to quickly and automatically create and align a set of homologous (and so alignable) contigs. (*A*) Shows the four lines of R code required for this analysis. (*B*) Shows that the input files can be split among many folders, and demonstrates the naming convention for input files. *Allolobophora chlorotica* is the root directory containing *ACHLO* and *RBNII* subdirectories. Sixteen ABIF files are distributed in these two subdirectories, and each of them is named with a contig name plus a direction suffix. (*C*) Shows a screenshot of the Shiny application that pops up when running the second line of R code in (*A*). The application allows users to access all reads and contigs through the navigation bar on the left. (*D*) and (*E*) are respectively the alignment and phylogenetic trees for the eight contigs created in this analysis.

To illustrate the capabilities of sangeranalyseR (fig. 1), we analyze data from eight annelid (*Allolobophora chlorotica*) samples downloaded from the Barcode Of Life Database (https://www.boldsystems.org/, last accessed February 22, 2021), each of which was sequenced with one forward and one reverse read. This is a subset of the example data included in the sangeranalyseR package. The entire analysis, including loading the data, trimming the reads, and assembling and aligning contigs, and producing a detailed interactive HTML report, can be completed with just four lines of R code (fig. 2*A*). Figure 2 shows the workflow including the R code (fig. 2*A*), the input data (fig. 2*B*), a screen capture from the Shiny application which allows users to adjust various settings in the analysis (fig. 2*C*), and the aligned contigs and their associated phylogenetic tree (fig. 2*D* and *E*). We discuss the workflow in more detail below.

### Preparing Input Files

sangeranalyseR needs two kinds of input information: Sanger sequencing reads in either ABIF or FASTA format, and information on how these reads should be grouped into contigs. The latter information can be provided in two ways. First, it can be provided implicitly with widely used naming conventions in which the start of each filename defines the contig-group, and the end of each filename defines whether each read is in the forward- or reverse-orientation. Second, the same information can be provided in comma-separated value (csv) file with three columns: The read name, whether the read is in the forward- or reverse-orientation, and the group to which the read should be assigned. In this demonstration analysis, input files are all in ABIF format and are stored in two subdirectories (fig. 2*B*). The filenames themselves are sufficient to determine how the reads should be grouped into contigs.

### Loading Reads into R Environment

In this example, sixteen reads are grouped into eight contigs containing one forward- and one reverse-read each. After grouping (determined using the regular expression in the loading command which parses the filename of each read), each read is trimmed, then reads are aligned into contigs and contigs are aligned to each other (e.g., fig. 2*D* and *E*). All results, including alignments, are stored inside the final *SangerAlignment* object. All these processes are initiated automatically by simply executing one line of R code (fig. 2*A*) that calls the *SangerAlignment* function. This function provides sensible defaults for all parameters, but also provides full flexibility by allowing users to alter each analysis parameter when calling the function. In this way, analyses are simple, flexible, and reproducible.

### Exploring the Data in the Shiny Application

After the *SangerAlignment* object is created, users can optionally explore the data (*third step* in fig. 1) by launching an interactive Shiny application with one line of R code which calls the *launchApp* function. The Shiny application provides an intuitive and interactive way to adjust parameters at all levels of the analysis, from the read to the assembled contig, and to view the read trimming, alignment of reads into contigs, alignment of contigs to each other (e.g., fig. 2*D*) and the Neighbor-Joining tree calculated from that alignment (fig. 2*E*). The various levels of the analysis are navigated via the navigation bar on the left of figure 2*C*. To facilitate reproducibility, once the necessary adjustments have been made, the user can save the new parameters and close the application with the “Save S4 instance” and “Close UI” buttons, respectively. When the new parameters are saved, the parameter values are stored in the *SangerAlignment* object ensuring that analyses are reproducible even if adjustments are made interactively in the Shiny application.

**Fig. 3.**
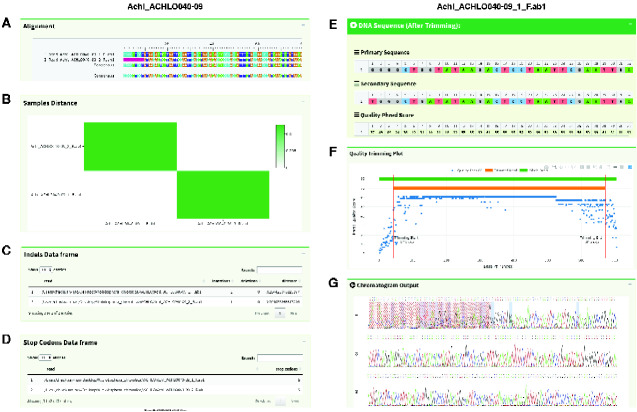
The Shiny application allows users to quickly interrogate contigs (left hand column) and individual reads (right hand column). For the analysis of a single contig (*A*) shows the alignment of reads and the consensus read; (*B*) is the heatmap showing the distance between the reads in the contig; and (*C*) and (*D*) are the data frames of indels and stop codons in the individual reads. For the analysis of a single read, (*E*) shows the trimmed primary sequence, secondary sequence, and the quality score for each nucleotide; (*F*) shows the interactive quality trimming plot with the trimming positions at 3′ and 5′ ends labeled with a red line, and the green bar and orange bar representing the extent of the untrimmed and trimmed read, respectively; (*G*) shows the chromatogram of the read with the trimmed portion hatched in red. The colors of A/T/C/G signal lines match the colors of nucleotides in (*E*). Colors in the Shiny application can be adjusted in the package to suit colorblind users.

Figure 3 demonstrates some of the information available at the level of individual contigs (fig. 3*A*–*D*) and reads (fig. 3*E*–*G*). At the level of the contig, users can view the alignment of all reads that form the contig (fig. 3*A*), the Hamming distances (number of differences) between all pairs of reads (fig. 3*B*), and tables of information on indels (fig. 3*C*) and stop codons (fig. 3*D*) contained in each read. The latter two tables are calculated only if the user provides an amino-acid reference sequence for their reads. At the level of the individual read, users can view the primary and secondary sequences that were called from the raw data in the ABIF file, the Phred quality score for each nucleotide, information on secondary peaks, and which bases in the read were trimmed (fig. 3*E* and *F*). Users can interactively adjust the parameters for calling secondary peaks and read trimming to assist with detecting and removing low-quality data (e.g., from sequencing mixtures of PCR products that produce many secondary peaks and/or for removing low-quality data from the ends of reads). Figure 3*G* shows the chromatogram, with the 3′ and 5′ end trimmed regions highlighted with red hatching. Both alignments and chromatograms can be set to display in colorblind-friendly palettes in sangeranalyseR.

sangernalyseR allows users to choose from two read trimming algorithms. The first one is the modified Mott trimming algorithm which is implemented in Phred (Ewing and Green 1998; Ewing et al. 1998), and BioPython (Cock et al. 2009). The second one is the sliding window trimming approach implemented in Trimmomatic (Bolger et al. 2014). The cutoff value for both algorithms can be adjusted with the “Trimming Cutoff” parameter in either R or Shiny applications.

### Writing Results to FASTA Files

After exploring the data in the Shiny application, users can output their read and contig sequences into FASTA files (*fourth step* in fig. 1). There are four output options including outputting all trimmed reads, consensus contigs, aligned consensus contigs, and all of the above.

### Generating Interactive HTML Report

Users can optionally create an interactive HTML report by running one line of R code which calls the *generateReport* function (*Fifth step* in fig. 1). Three levels of results, reads, contigs, and alignment, are created and stored hierarchically in a way that mirrors the output of the Shiny application (though does not allow for parameters to be changed).

## Discussion

sangeranalyseR provides a simple and powerful suite of functions to process Sanger sequencing data in R. It automates most standard tasks with sensible but adjustable default parameter values which can be accessed either via the command-line in R or through an interactive Shiny interface. It has thorough documentation in both a vignette and online using ReadtheDocs (https://sangeranalyser.readthedocs.io, last accessed February 22, 2021; Supplementary Material online). It also includes a large suite of unit tests managed by testthat (Wickham 2011), and automated by Travis CI (Travis CI Builder Team 2012). sangeranalyseR is open-source and maintained on Github: http://github.com/roblanf/sangeranalyseR (last accessed February 22, 2021).

The standardized HTML reports produced by sangeranalyseR will assist with the communication and interpretation of results both within a single project, and across multiple projects. The fact that sangeranalyseR allows for reproducible workflows will allow research teams to more easily share and build on their own and each others’ analyses, and to rapidly repeat similar analyses across multiple data sets. sangeranalyseR allows users to query and analyze their data within the R environment, thus exposing the results of their analysis to the huge range of other R packages for bioinformatics. sangeranalyseR outputs all data in the widely-used FASTA format to ensure interoperability with bioinformatic tools outside the R environment.

One key limitation of sangeranalyseR is that it does not support editing of individual bases in each read inside the Shiny application. This is primarily due to the limitations of the R environment. In principle, individual bases of individual reads can be edited by simply editing the “BioStrings” representation of each read, but we appreciate that this is not a very practical solution for most users. Rather, most users who want to edit individual bases tend to want an interface that allows them to do this while viewing the chromatogram. To alleviate this as much as possible, we note that many other applications support such editing, and that edited reads from those applications can be input to sangeranalyseR using the FASTA file input option. MEGA (Kumar et al. 2018) and BioEdit (Hall 1999) are both free and widely used multi-platform software tools that can be used to edit chromatograms from AB1 files. The edited reads can be saved in FASTA format and then loaded into sangeranalyseR.

## Materials and Methods

### Software Implementation

sangeranalyseR is implemented as an R package with three well-designed S4 classes, *SangerRead*, *SangerContig*, and *SangerAlignment*, integrated with Shiny applications and RMarkdown. Here, we give short descriptions of the three S4 classes. *SangerRead* extends the *sangerseq* S4 class from sangerseqR (Hill et al. 2014) and contains the chromatogram as well as the quality trimming parameters and results; *SangerContig* stores four things: A contig, the forward and reverse reads that were used to create it with the *ConsensusSequence* function in DECIPHER, a dendrogram created with the *IdClusters* function, and a data frame with information on indels and stop codons. If a reference amino-acid sequence is provided, then frameshift errors in protein-coding sequences can be corrected. *SangerAlignment* contains three things: A list of all the contigs, an alignment of the contigs, and a phylogenetic tree of the aligned contigs. The alignment and phylogenetic tree are included for quality control purposes, and are not intended as best-practice alignments or trees for downstream analyses. Contigs are aligned using the *AlignSeqs* or the *AlignTranslation* functions from the DECIPHER package, and the phylogenetic tree is inferred by ape’s (Paradis and Schliep 2019) *bionj* function. All reads and consensus sequences in sangeranalyseR are stored as *DNAString* objects from the Biostrings (Pagès et al. 2020) package.

### Input Files

sangeranalyseR is an R package that accepts input files in ABIF or FASTA formats. ABIF format is a binary data storage file type storing sequencing information generated by Applied Biosystems machines, and FASTA format is a text-based file type storing nucleotide sequences. Since the FASTA format doesn’t contain the raw data used to call base sequences, when FASTA files are used as input some features like trimming, chromatogram plotting, and base calling are not available. In creating *SangerContig* and *SangerAlignment* objects, users can choose either regular expression matching or use a separate csv input file to categorize reads into forward and reverse directions and into different contig groups.

### Shiny Application Interface

One of sangeranalyseR’s most powerful features is that it integrates two Shiny applications inside the R package. The following is the design of how Shiny applications are embedded in the R package: The input of *lauchApp* function, *SangerContig* or *SangerAlignment* S4 object, is passed into the Shiny application through *shinyOptions* function. After manually tuning parameters, the updated S4 object is saved as an RDA file in a temporary directory and can be loaded by the *readRDS* function back into the R environment. Inside the Shiny application, user inputs are created with the *reactiveValues* function, and HTML widgets are monitored by the *observeEvent* function to make Shiny applications dynamic and real-time to users; the shinydashboard (Ribeiro 2018) and shinyWidgets (Yihui 2019) R packages are used to create the modularized user interface; shinycssloaders (Sali 2017) offers loading animations whereas dynamic widgets are reloading; and shinyjs (Attali 2020) creates a Javascript interface to make applications more interactive. DNA and amino-acid sequences are displayed with *excelTable* in excelR (Ribeiro 2018); the interactive trimming plot and heatmaps are created with plotly (Plotly Technologies Inc. 2015); dataframe results like indels and stop codons are displayed by *datatable* in DT (Yihui 2019).

### Output Files

sangeranalyseR lets users output analysis results into FASTA files and interactive HTML reports. These features use the *writeXStringSet* function in Biostrings (Pagès et al. 2020) to write FASTA files, and the *render* function in rmarkdown (Allaire et al. 2020; Xie et al. 2018, 2020) is recursively called to hierarchically knit *SangerRead*, *SangerContig*, and *SangerAlignment* HTML reports.

## Supplementary Material

Supplementary data are available at *Genome Biology and Evolution* online.

## Supplementary Material

evab028_Supplementary_Data

## Data Availability

The ABIF files for the samples underlying this article are available in Barcode of Life database http://www.boldsystems.org/ (last accessed February 22, 2021) with sequence IDs ACHLO006-09, ACHLO007-09, ACHLO040-09, ACHLO041-09, RBNII384-13, RBNII395-13, RBNII396-13, RBNII397-13, BBDCN941-10, BBDEE689-10, PHDIP946-11, TDWGB557-10, and TDWGB669-10. The R package source code is available on Github: https://github.com/roblanf/sangeranalyseR (last accessed February 22, 2021) and Bioconductor: https://www.bioconductor.org/packages/release/bioc/html/sangeranalyseR.html (last accessed February 22, 2021).
