# Supplementary material for: sangeranalyseR: Simple and Interactive Processing of Sanger Sequencing Data in R
Source: Genome Biol Evol. 2021 Feb 16;13(3):evab028. doi: 10.1093/gbe/evab028 (PMC7939931; doi:10.1093/gbe/evab028)
Supplement: evab028_Supplementary_Data [file evab028_supplementary_data.zip › sangeranalyseR_manual.pdf]

Package ‘sangeranalyseR’

July 27, 2020

Type Package

Title sangeranalyseR: a suite of functions for the analysis of Sanger sequence data in R

Version 0.99.25

Date 2020-04-22

Author Rob Lanfear <rob.lanfear@gmail.com>, Kuan-Hao Chao <ntueeb05howard@gmail.com>

Maintainer Kuan-Hao Chao <ntueeb05howard@gmail.com>

biocViews Genetics, Alignment, Sequencing, SangerSeq, Preprocessing, QualityControl, Visualization, GUI

Description This package builds on sangerseqR to allow users to create contigs from collections of Sanger sequencing reads. It provides a wide range of options for a number of commonly-performed actions including read trimming, detecting secondary peaks, and detecting indels using a reference sequence. All parameters can be adjusted interactively either in R or in the associated Shiny applications. There is extensive online documentation, and the package can outputs detailed HTML reports, including chromatograms.

License GPL-2

Encoding UTF-8

Depends R (>= 4.0.0), stringr, ape, Biostrings, DECIPHER, parallel, reshape2, phangorn, sangerseqR, gridExtra, shiny, shinydashboard, shinyjs, data.table, plotly, DT, zeallot, excelR, shinycssloaders, ggdendro, shinyWidgets, openxlsx, tools, rmarkdown, kableExtra, seqinr, BiocStyle, logger

RoxygenNote 6.1.1

VignetteBuilder knitr

Suggests knitr, testthat (>= 2.1.0)

Collate 'AllGenerics.R' 'ClassChromatogramParam.R' 'ClassQualityReport.R' 'ClassSangerRead.R' 'ClassSangerAlignment.R' 'ClassSangerContig.R' 'Constructors.R' 'LoadMessage.R' 'MethodSangerAlignment.R' 'MethodSangerContig.R' 'MethodSangerRead.R' 'MethodShared.R' 'MethodsQualityReport.R' 'ShinySangerAlignmentServer.R' 'ShinySangerAlignmentUI.R' 'ShinySangerContigServer.R' 'ShinySangerContigUI.R' 'ShinyServerModule.R' 'UtilitiesFunc.R' 'UtilitiesFuncInputChecker.R' 'data.R' 'sangeranalyseR_package.R' 'sangeranalyseR_show_method.R'

git_url https://git.bioconductor.org/packages/sangeranalyseR

git_branch master

git_last_commit 17daa36

git_last_commit_date 2020-06-29

Date/Publication 2020-07-26

R topics documented:

ChromatogramParam-class . . . 3
generateReport . . . 3
generateReportSA . . . 4
generateReportSC . . . 5
generateReportSR . . . 6
launchApp . . . 6
launchAppSA . . . 7
launchAppSC . . . 8
MakeBaseCalls . . . 8
qualityBasePlot . . . 9
QualityReport-class . . . 9
QualityReport-class-qualityBasePlot . . . 10
QualityReport-class-updateQualityParam . . . 11
qualityReportData . . . 12
readTable . . . 12
SangerAlignment . . . 13
SangerAlignment-class . . . 16
SangerAlignment-class-generateReportSA . . . 18
SangerAlignment-class-launchAppSA . . . 19
SangerAlignment-class-updateQualityParam . . . 19
SangerAlignment-class-writeFastaSA . . . 20
sangerAlignmentData . . . 21
sangeranalyseR . . . 21
SangerContig . . . 22
SangerContig-class . . . 24
SangerContig-class-generateReportSC . . . 27
SangerContig-class-launchAppSC . . . 28
SangerContig-class-updateQualityParam . . . 29
SangerContig-class-writeFastaSC . . . 30
sangerContigData . . . 31
SangerRead . . . 31
SangerRead-class . . . 33
SangerRead-class-generateReportSR . . . 35
SangerRead-class-MakeBaseCalls . . . 36
SangerRead-class-qualityBasePlot . . . 36
SangerRead-class-readTable . . . 37
SangerRead-class-updateQualityParam . . . 38
SangerRead-class-writeFastaSR . . . 39
sangerReadFData . . . 39
updateQualityParam . . . 40
writeFasta . . . 41
writeFastaSA . . . 42

writeFastaSC 43
writeFastaSR 43

Index **45**

`ChromatogramParam-class` *ChromatogramParam*

Description

An S4 class storing chromatogram related inputs in a SangerRead S4 object.

Slots

`baseNumPerRow` It defines maximum base pairs in each row. The default value is `100`.

`heightPerRow` It defines the height of each row in chromatogram. The default value is `200`.

`signalRatioCutoff` The ratio of the height of a secondary peak to a primary peak. Secondary peaks higher than this ratio are annotated. Those below the ratio are excluded. The default value is `0.33`.

`showTrimmed` The logical value storing whether to show trimmed base pairs in chromatogram. The default value is `TRUE`.

Author(s)

Kuan-Hao Chao

Examples

```
Chromatogram <- new("ChromatogramParam",
                    baseNumPerRow     = 100,
                    heightPerRow      = 200,
                    signalRatioCutoff  = 0.33,
                    showTrimmed       = TRUE)
```

`generateReport` *Method generateReport*

Description

A method which generates final reports of the SangerRead, SangerContig, and SangerAlignment instance.

Usage

```
generateReport(object, outputDir = NULL, includeSangerContig = TRUE,
  includeSangerRead = TRUE, ...)
```

Arguments

`object`	A SangerRead, SangerContig, or SangerAlignment S4 instance.
`outputDir`	The output directory of the generated HTML report.
`includeSangerContig`	The parameter that decides whether to include SangerContig level report. The value is `TRUE` or `FALSE` and the default is `TRUE`.
`includeSangerRead`	The parameter that decides whether to include SangerRead level report. The value is `TRUE` or `FALSE` and the default is `TRUE`.
`...`	Further generateReportSR, generateReportSC, and generateReportSA related parameters.

Value

A `SangerRead`, `SangerContig`, or `SangerAlignment` object.

Author(s)

Kuan-Hao Chao

Examples

```
data(sangerReadFData)
data(sangerContigData)
data(sangerAlignmentData)
## Not run:
generateReport(sangerReadFData)
generateReport(sangerContigData)
generateReport(sangerAlignmentData)
## End(Not run)
```

`generateReportSA` *Method generateReportSA*

Description

Method generateReportSA

Usage

```
generateReportSA(object, outputDir = NULL, includeSangerContig = TRUE,
  includeSangerRead = TRUE, ...)
```

Arguments

`object`	A SangerAlignment S4 instance.
`outputDir`	The output directory of the generated HTML report.
`includeSangerContig`	The parameter that decides whether to include SangerContig level report. The value is `TRUE` or `FALSE` and the default is `TRUE`.

includeSangerRead
: The parameter that decides whether to include SangerRead level report. The value is TRUE or FALSE and the default is TRUE.

... Further generateReportSA-related parameters.

Value

The output absolute path to the SangerAlignment's HTML file.

Examples

```
data(sangerAlignmentData)
## Not run:
generateReportSA(sangerAlignmentData)
## End(Not run)
```

---

generateReportSC *Method generateReportSC*

Description

Method generateReportSC

Usage

```
generateReportSC(object, outputDir = NULL, includeSangerRead = TRUE,
  ...)
```

Arguments

object A SangerContig S4 instance.

outputDir The output directory of the generated HTML report.

includeSangerRead
: The parameter that decides whether to include SangerRead level report. The value is TRUE or FALSE and the default is TRUE.

... Further generateReportSC-related parameters.

Value

The output absolute path to the SangerContig's HTML file.

Examples

```
data(sangerContigData)
## Not run:
generateReportSC(sangerContigData)
## End(Not run)
```

generateReportSR *Method generateReportSR*

Description

Method generateReportSR

Usage

```
generateReportSR(object, outputDir = NULL, ...)
```

Arguments

`object`	A SangerRead S4 instance.
`outputDir`	The output directory of the generated HTML report.
`...`	Further generateReportSR-related parameters.

Value

The output absolute path to the SangerRead's HTML file.

Examples

```
data(sangerReadFData)
## Not run:
generateReportSR(sangerReadFData)
## End(Not run)
```

launchApp *Method launchApp*

Description

A method which launches Shiny application of the SangerContig and SangerAlignment instance.

Usage

```
launchApp(object, outputDir = NULL)
```

Arguments

`object`	A SangerContig or SangerAlignment S4 instance.
`outputDir`	The output directory of the saved new SangerContig or SangerAlignment S4 instance.

Value

A `SangerContig` or `SangerAlignment` object.

Author(s)

Kuan-Hao Chao

Examples

```
data(sangerContigData)
data(sangerAlignmentData)
## Not run:
launchApp(sangerContigData)
launchApp(sangerAlignmentData)
## End(Not run)
```

`launchAppSA` *Method launchAppSA*

Description

Method launchAppSA

Usage

```
launchAppSA(object, outputDir = NULL)
```

Arguments

`object`	A SangerAlignment S4 instance.
`outputDir`	The output directory of the saved new SangerAlignment S4 instance.

Value

A `shiny.appobj` object.

Examples

```
data(sangerAlignmentData)
## Not run:
launchAppSA(sangerAlignmentData)
## End(Not run)
```

launchAppSC — *Method launchAppSC*

Description

Method launchAppSC

Usage

```
launchAppSC(object, outputDir = NULL)
```

Arguments

object	A SangerContig S4 instance.
outputDir	The output directory of the saved new SangerContig S4 instance.

Value

A `shiny.appobj` object.

Examples

```
data(sangerContigData)
## Not run:
launchAppSC(sangerContigData)
## End(Not run)
```

MakeBaseCalls — *Method MakeBaseCalls*

Description

Method MakeBaseCalls

Usage

```
MakeBaseCalls(object, signalRatioCutoff = 0.33)
```

Arguments

object	A SangerRead S4 instance.
signalRatioCutoff	The ratio of the height of a secondary peak to a primary peak. Secondary peaks higher than this ratio are annotated. Those below the ratio are excluded. The default value is `0.33`.

Value

A `SangerRead` instance.

Examples

```
data(sangerReadFData)
MakeBaseCalls(sangerReadFData, signalRatioCutoff = 0.22)
```

`qualityBasePlot` *Method qualityBasePlot*

Description

Method qualityBasePlot

Usage

```
qualityBasePlot(object)
```

Arguments

`object`	A QualityReport or SangerRead S4 instance

Value

A quality plot.

Examples

```
data(qualityReportData)
data(sangerReadFData)
qualityBasePlot(qualityReportData)
qualityBasePlot(sangerReadFData)
```

`QualityReport-class` *QualityReport*

Description

An S4 class storing quality related inputs and results in a SangerRead S4 object.

Slots

`TrimmingMethod` The read trimming method for this SangerRead. The value must be `"M1"` (the default) or `'M2'`.

`M1TrimmingCutoff` The trimming cutoff for the Method 1. If `TrimmingMethod` is `"M1"`, then the default value is `0.0001`. Otherwise, the value must be `NULL`.

`M2CutoffQualityScore` The trimming cutoff quality score for the Method 2. If `TrimmingMethod` is `'M2'`, then the default value is `20`. Otherwise, the value must be `NULL`. It works with `M2SlidingWindowSize`.

`M2SlidingWindowSize` The trimming sliding window size for the Method 2. If `TrimmingMethod` is `'M2'`, then the default value is `10`. Otherwise, the value must be `NULL`. It works with `M2CutoffQualityScore`.

qualityPhredScores The Phred quality scores of each base pairs after base calling.

qualityBaseScores The probability of incorrect base call of each base pairs. They are calculated from qualityPhredScores.

rawSeqLength The number of nucleotides of raw primary DNA sequence.

trimmedSeqLength The number of nucleotides of trimeed primary DNA sequence.

trimmedStartPos The base pair index of trimming start point from 5' end of the sequence.

trimmedFinishPos The base pair index of trimming finish point from 3' end of the sequence.

rawMeanQualityScore The mean quality score of the primary sequence after base calling. In other words, it is the mean of qualityPhredScores.

trimmedMeanQualityScore The mean quality score of the trimmed primary sequence after base calling.

rawMinQualityScore The minimum quality score of the primary sequence after base calling.

trimmedMinQualityScore The minimum quality score of the trimmed primary sequence after base calling.

remainingRatio The remaining sequence length ratio after trimming.

Author(s)

Kuan-Hao Chao

Examples

```
inputFilesPath <- system.file("extdata/", package = "sangeranalyseR")
A_chloroticaFFN <- file.path(inputFilesPath,
                             "Allolobophora_chlorotica",
                             "ACHLO",
                             "Achl_ACHLO006-09_1_F.ab1")
sangerReadF <- new("SangerRead",
                   inputSource          = "ABIF",
                   readFeature          = "Forward Read",
                   readFileName         = A_chloroticaFFN,
                   geneticCode          = GENETIC_CODE,
                   TrimmingMethod       = "M1",
                   M1TrimmingCutoff     = 0.0001,
                   M2CutoffQualityScore  = NULL,
                   M2SlidingWindowSize   = NULL,
                   baseNumPerRow        = 100,
                   heightPerRow         = 200,
                   signalRatioCutoff     = 0.33,
                   showTrimmed          = TRUE)
"@"(sangerReadF, QualityReport)
```

QualityReport-class-qualityBasePlot *qualityBasePlot*

Description

A QualityReport method which creates quality base interactive plot.

Usage

```
## S4 method for signature 'QualityReport'
qualityBasePlot(object)
```

Arguments

`object`	A QualityReport S4 instance.

Value

A quality plot.

Examples

```
data("qualityReportData")
## Not run:
qualityBasePlot(qualityReportData)
## End(Not run)
```

QualityReport-class-updateQualityParam
updateQualityParam

Description

A QualityReport method which updates quality base interactive plot.

Usage

```
## S4 method for signature 'QualityReport'
updateQualityParam(object,
  TrimmingMethod = "M1", M1TrimmingCutoff = 1e-04,
  M2CutoffQualityScore = NULL, M2SlidingWindowSize = NULL)
```

Arguments

`object`	A QualityReport S4 instance.
`TrimmingMethod`	The read trimming method for this SangerRead. The value must be `"M1"` (the default) or `'M2'`.
`M1TrimmingCutoff`	The trimming cutoff for the Method 1. If `TrimmingMethod` is `"M1"`, then the default value is `0.0001`. Otherwise, the value must be `NULL`.
`M2CutoffQualityScore`	The trimming cutoff quality score for the Method 2. If `TrimmingMethod` is `'M2'`, then the default value is `20`. Otherwise, the value must be `NULL`. It works with `M2SlidingWindowSize`.
`M2SlidingWindowSize`	The trimming sliding window size for the Method 2. If `TrimmingMethod` is `'M2'`, then the default value is `10`. Otherwise, the value must be `NULL`. It works with `M2CutoffQualityScore`.

Value

A QualityReport instance.

Examples

```
data("qualityReportData")
updateQualityParam(qualityReportData,
                   TrimmingMethod         = "M2",
                   M1TrimmingCutoff       = NULL,
                   M2CutoffQualityScore   = 30,
                   M2SlidingWindowSize    = 15)
```

`qualityReportData` *QualityReport instance*

Description

QualityReport instance

Usage

```
data(qualityReportData)
```

Author(s)

Kuan-Hao Chao

`readTable` *Method readTable*

Description

Method readTable

Usage

```
readTable(object, indentation, ...)
```

Arguments

`object`	A SangerRead, SangerContig, or SangerAlignment S4 instance.
`indentation`	The indentation for different level printing
`...`	Further generateReportSR-related parameters.

Value

None.

Examples

```
data(sangerReadFData)
data(sangerContigData)
data(sangerAlignmentData)
## Not run:
readTable(sangerReadFData)
readTable(sangerContigData)
readTable(sangerAlignmentData)

## End(Not run)
```

SangerAlignment *SangerAlignment*

Description

the wrapper function for SangerAlignment

Usage

```
SangerAlignment(inputSource = "ABIF", fastaFileName = "",
  namesConversionCSV = NULL, parentDirectory = "",
  suffixForwardRegExp = "_F.ab1", suffixReverseRegExp = "_R.ab1",
  TrimmingMethod = "M1", M1TrimmingCutoff = 1e-04,
  M2CutoffQualityScore = NULL, M2SlidingWindowSize = NULL,
  baseNumPerRow = 100, heightPerRow = 200, signalRatioCutoff = 0.33,
  showTrimmed = TRUE, refAminoAcidSeq = "", minReadsNum = 2,
  minReadLength = 20, minFractionCall = 0.5, maxFractionLost = 0.5,
  geneticCode = GENETIC_CODE, acceptStopCodons = TRUE,
  readingFrame = 1, minFractionCallSA = 0.5, maxFractionLostSA = 0.5,
  processorsNum = NULL)
```

Arguments

`inputSource` The input source of the raw file. It must be "ABIF" or "FASTA". The default value is "ABIF".

`fastaFileName` If inputSource is "FASTA", then this value has to be the name of the FASTA file; if inputSource is "ABIF", then this value is "" by default.

`namesConversionCSV`
The file path to the CSV file that provides read names that follow the naming regulation. If inputSource is "FASTA", then users need to prepare the csv file or make sure the original names inside FASTA file are valid; if inputSource is "ABIF", then this value is NULL by default.

`parentDirectory`
The parent directory of all of the reads contained in ABIF format you wish to analyse. In SangerAlignment, all reads in subdirectories will be scanned recursively.

`suffixForwardRegExp`
The suffix of the filenames for forward reads in regular expression, i.e. reads that do not need to be reverse-complemented. For forward reads, it should be "_F.ab1".

`suffixReverseRegExp`
: The suffix of the filenames for reverse reads in regular expression, i.e. reads that need to be reverse-complemented. For revcerse reads, it should be `"_R.ab1"`.

`TrimmingMethod`
: TrimmingMethod The read trimming method for this SangerRead. The value must be `"M1"` (the default) or `'M2'`.

`M1TrimmingCutoff`
: The trimming cutoff for the Method 1. If `TrimmingMethod` is `"M1"`, then the default value is `0.0001`. Otherwise, the value must be `NULL`.

`M2CutoffQualityScore`
: The trimming cutoff quality score for the Method 2. If `TrimmingMethod` is `'M2'`, then the default value is `20`. Otherwise, the value must be `NULL`. It works with `M2SlidingWindowSize`.

`M2SlidingWindowSize`
: The trimming sliding window size for the Method 2. If `TrimmingMethod` is `'M2'`, then the default value is `10`. Otherwise, the value must be `NULL`. It works with `M2CutoffQualityScore`.

`baseNumPerRow`
: It defines maximum base pairs in each row. The default value is `100`.

`heightPerRow`
: It defines the height of each row in chromatogram. The default value is `200`.

`signalRatioCutoff`
: The ratio of the height of a secondary peak to a primary peak. Secondary peaks higher than this ratio are annotated. Those below the ratio are excluded. The default value is `0.33`.

`showTrimmed`
: The logical value storing whether to show trimmed base pairs in chromatogram. The default value is `TRUE`.

`refAminoAcidSeq`
: An amino acid reference sequence supplied as a string or an AAString object. If your sequences are protein-coding DNA seuqences, and you want to have frameshifts automatically detected and corrected, supply a reference amino acid sequence via this argument. If this argument is supplied, the sequences are then kept in frame for the alignment step. Fwd sequences are assumed to come from the sense (i.e. coding, or "+") strand. The default value is `""`.

`minReadsNum`
: The minimum number of reads required to make a consensus sequence, must be 2 or more. The default value is `2`.

`minReadLength`
: Reads shorter than this will not be included in the readset. The default `20` means that all reads with length of 20 or more will be included. Note that this is the length of a read after it has been trimmed.

`minFractionCall`
: Minimum fraction of the sequences required to call a consensus sequence for SangerContig at any given position (see the ConsensusSequence() function from DECIPHER for more information). Defaults to 0.75 implying that 3/4 of all reads must be present in order to call a consensus.

`maxFractionLost`
: Numeric giving the maximum fraction of sequence information that can be lost in the consensus sequence for SangerContig (see the ConsensusSequence() function from DECIPHER for more information). Defaults to 0.5, implying that each consensus base can ignore at most 50 percent of the information at a given position.

`geneticCode`
: Named character vector in the same format as `GENETIC_CODE` (the default), which represents the standard genetic code. This is the code with which the

function will attempt to translate your DNA sequences. You can get an appropriate vector with the getGeneticCode() function. The default is the standard code.

`acceptStopCodons`
: The logical value `TRUE` or `FALSE`. `TRUE` (the defualt): keep all reads, regardless of whether they have stop codons; `FALSE`: reject reads with stop codons. If `FALSE` is selected, then the number of stop codons is calculated after attempting to correct frameshift mutations (if applicable).

`readingFrame`
: `1`, `2`, or `3`. Only used if `accept.stop.codons == FALSE`. This specifies the reading frame that is used to determine stop codons. If you use a `refAminoAcidSeq`, then the frame should always be `1`, since all reads will be shifted to frame 1 during frameshift correction. Otherwise, you should select the appropriate reading frame.

`minFractionCallSA`
: Minimum fraction of the sequences required to call a consensus sequence for SangerAlignment at any given position (see the ConsensusSequence() function from DECIPHER for more information). Defaults to 0.75 implying that 3/4 of all reads must be present in order to call a consensus.

`maxFractionLostSA`
: Numeric giving the maximum fraction of sequence information that can be lost in the consensus sequence for SangerAlignment (see the ConsensusSequence() function from DECIPHER for more information). Defaults to 0.5, implying that each consensus base can ignore at most 50 percent of the information at a given position.

`processorsNum`
: The number of processors to use, or NULL (the default) for all available processors.

Value

A SangerAlignment instance.

Author(s)

Kuan-Hao Chao

Examples

```
rawDataDir <- system.file("extdata", package = "sangeranalyseR")
parentDir <- file.path(rawDataDir, "Allolobophora_chlorotica", "RBNII")
suffixForwardRegExp <- "_[0-9]*_F.ab1"
suffixReverseRegExp <- "_[0-9]*_R.ab1"
sangerAlignment <- SangerAlignment(
                       inputSource            = "ABIF",
                       parentDirectory       = parentDir,
                       suffixForwardRegExp   = suffixForwardRegExp,
                       suffixReverseRegExp   = suffixReverseRegExp,
               refAminoAcidSeq = "SRQWLFSTNHKDIGTLYFIFGAWAGMVGTSLSILIRAELGHPGALIGDDQIYNVIVTAHAFIMIFFMVMPIMI
                       TrimmingMethod        = "M1",
                       M1TrimmingCutoff      = 0.0001,
                       M2CutoffQualityScore  = NULL,
                       M2SlidingWindowSize   = NULL,
                       baseNumPerRow         = 100,
                       heightPerRow          = 200,
                       signalRatioCutoff     = 0.33,
```

```
showTrimmed          = TRUE,
processorsNum        = 2)
```

SangerAlignment-class *SangerAlignment*

Description

An S4 class containing SangerContigs lists and contigs alignment results which corresponds to a final alignment in Sanger sequencing.

Slots

`inputSource` The input source of the raw file. It must be `"ABIF"` or `"FASTA"`. The default value is `"ABIF"`.

`fastaFileName` If `inputSource` is `"FASTA"`, then this value has to be the name of the FASTA file; if `inputSource` is `"ABIF"`, then this value is `""` by default.

`namesConversionCSV` The file path to the CSV file that provides read names that follow the naming regulation. If `inputSource` is `"FASTA"`, then users need to prepare the csv file or make sure the original names inside FASTA file are valid; if `inputSource` is `"ABIF"`, then this value is `NULL` by default.

`parentDirectory` If `inputSource` is `"ABIF"`, then this value is the path of the parent directory storing all reads in ABIF format you wish to analyse and cannot be NULL. In SangerAlignment, all reads in subdirectories will be scanned recursively. If `inputSource` is `"FASTA"`, then this value is `NULL` by default.

`suffixForwardRegExp` The suffix of the filenames for forward reads in regular expression, i.e. reads that do not need to be reverse-complemented. For forward reads, it should be `"_F.ab1"`.

`suffixReverseRegExp` The suffix of the filenames for reverse reads in regular expression, i.e. reads that need to be reverse-complemented. For revcerse reads, it should be `"_R.ab1"`.

`trimmingMethodSA` The read trimming method for all SangerRead S4 instances in SangerAlignment. The value must be `"M1"` (the default) or `'M2'`. All SangerReads must have the same trimming method.

`minFractionCallSA` Minimum fraction of the sequences required to call a consensus sequence for SangerAlignment at any given position (see the ConsensusSequence() function from DECIPHER for more information). Defaults to 0.75 implying that 3/4 of all reads must be present in order to call a consensus.

`maxFractionLostSA` Numeric giving the maximum fraction of sequence information that can be lost in the consensus sequence for SangerAlignment (see the ConsensusSequence() function from DECIPHER for more information). Defaults to 0.5, implying that each consensus base can ignore at most 50 percent of the information at a given position.

`geneticCode` Named character vector in the same format as `GENETIC_CODE` (the default), which represents the standard genetic code. This is the code with which the function will attempt to translate your DNA sequences. You can get an appropriate vector with the getGeneticCode() function. The default is the standard code.

`refAminoAcidSeq` An amino acid reference sequence supplied as a string or an AAString object. If your sequences are protein-coding DNA seuqences, and you want to have frameshifts automatically detected and corrected, supply a reference amino acid sequence via this argument. If this argument is supplied, the sequences are then kept in frame for the alignment step. Fwd sequences are assumed to come from the sense (i.e. coding, or "+") strand. The default value is `""`.

`contigList` A list storing all SangerContigs S4 instances.

`contigsConsensus` The consensus read of all SangerContig S4 instances in DNAString object.

`contigsAlignment` The alignment of all SangerContig S4 instances with the called consensus sequence in DNAStringSet object. Users can use BrowseSeqs() to view the alignment.

`contigsTree` A phylo instance returned by bionj function in ape package. It can be used to draw the tree.

Author(s)

Kuan-Hao Chao

Examples

```
## Input From ABIF file format (Regex)
rawDataDir <- system.file("extdata", package = "sangeranalyseR")
parentDir <- file.path(rawDataDir, 'Allolobophora_chlorotica', 'ACHLO')
suffixForwardRegExp <- "_[0-9]*_F.ab1"
suffixReverseRegExp <- "_[0-9]*_R.ab1"
sangerAlignment <- new("SangerAlignment",
                       inputSource           = "ABIF",
                       parentDirectory       = parentDir,
                       suffixForwardRegExp   = suffixForwardRegExp,
                       suffixReverseRegExp   = suffixReverseRegExp,
               refAminoAcidSeq = "SRQWLFSTNHKDIGTLYFIFGAWAGMVGTSLSILIRAELGHPGALIGDDQIYNVIVTAHAFIMIFFMVMPIMI
                       TrimmingMethod        = "M1",
                       M1TrimmingCutoff      = 0.0001,
                       M2CutoffQualityScore  = NULL,
                       M2SlidingWindowSize   = NULL,
                       baseNumPerRow         = 100,
                       heightPerRow          = 200,
                       signalRatioCutoff     = 0.33,
                       showTrimmed           = TRUE,
                       processorsNum         = 2)

## Input From ABIF file format (Csv three column)
rawDataDir <- system.file("extdata", package = "sangeranalyseR")
parentDir <- file.path(rawDataDir, 'Allolobophora_chlorotica', 'ACHLO')
namesConversionCSV <- file.path(rawDataDir, "ab1", "SangerAlignment",
"names_conversion.csv")
sangerAlignment <- new("SangerAlignment",
                       inputSource           = "ABIF",
                       parentDirectory       = parentDir,
                       namesConversionCSV    = namesConversionCSV,
               refAminoAcidSeq = "SRQWLFSTNHKDIGTLYFIFGAWAGMVGTSLSILIRAELGHPGALIGDDQIYNVIVTAHAFIMIFFMVMPIMI
                       TrimmingMethod        = "M1",
                       M1TrimmingCutoff      = 0.0001,
                       M2CutoffQualityScore  = NULL,
                       M2SlidingWindowSize   = NULL,
                       baseNumPerRow         = 100,
                       heightPerRow          = 200,
                       signalRatioCutoff     = 0.33,
                       showTrimmed           = TRUE,
                       processorsNum         = 2)

## Input From FASTA file format (No Csv - Regex)
rawDataDir <- system.file("extdata", package = "sangeranalyseR")
```

```
fastaFN <- file.path(rawDataDir, "fasta",
                     "SangerAlignment", "Sanger_all_reads.fa")
suffixForwardRegExpFa <- "_[0-9]*_F$"
suffixReverseRegExpFa <- "_[0-9]*_R$"
sangerAlignmentFa <- new("SangerAlignment",
                         inputSource          = "FASTA",
                         fastaFileName        = fastaFN,
                         suffixForwardRegExp  = suffixForwardRegExpFa,
                         suffixReverseRegExp  = suffixReverseRegExpFa,
                 refAminoAcidSeq = "SRQWLFSTNHKDIGTLYFIFGAWAGMVGTSLSILIRAELGHPGALIGDDQIYNVIVTAHAFIMIFFMVMPI[illegible]
                         processorsNum        = 2)

## Input From FASTA file format (Csv three column method)
rawDataDir <- system.file("extdata", package = "sangeranalyseR")
fastaFN <- file.path(rawDataDir, "fasta",
                     "SangerAlignment", "Sanger_all_reads.fa")
namesConversionCSV <- file.path(rawDataDir, "fasta",
                                "SangerAlignment", "names_conversion.csv")
sangerAlignmentFa <- new("SangerAlignment",
                         inputSource          = "FASTA",
                         fastaFileName        = fastaFN,
                         namesConversionCSV   = namesConversionCSV,
                 refAminoAcidSeq = "SRQWLFSTNHKDIGTLYFIFGAWAGMVGTSLSILIRAELGHPGALIGDDQIYNVIVTAHAFIMIFFMVMPI[illegible]
                         processorsNum        = 2)
```

`SangerAlignment-class-generateReportSA`
generateReportSA

Description

A SangerAlignment method which generates final reports of the SangerContig instance.

Usage

```
## S4 method for signature 'SangerAlignment'
generateReportSA(object, outputDir,
  includeSangerContig = TRUE, includeSangerRead = TRUE)
```

Arguments

`object`	A SangerAlignment S4 instance.
`outputDir`	The output directory of the generated HTML report.
`includeSangerContig`	The parameter that decides whether to include SangerContig level report. The value is `TRUE` or `FALSE` and the default is `TRUE`.
`includeSangerRead`	The parameter that decides whether to include SangerRead level report. The value is `TRUE` or `FALSE` and the default is `TRUE`.

Value

The output absolute path to the SangerAlignment's HTML file.

Examples

```
data("sangerAlignmentData")
## Not run:
generateReportSA(sangerAlignmentData)
## End(Not run)
```

SangerAlignment-class-launchAppSA
launchAppSA

Description

A SangerAlignment method which launches Shiny app for SangerAlignment instance.

Usage

```
## S4 method for signature 'SangerAlignment'
launchAppSA(object, outputDir = NULL)
```

Arguments

object	A SangerAlignment S4 instance.
outputDir	The output directory of the saved new SangerContig S4 instance.

Value

A shiny.appobj object.

Examples

```
data("sangerAlignmentData")
RShinySA <- launchAppSA(sangerAlignmentData)
```

SangerAlignment-class-updateQualityParam
updateQualityParam

Description

A SangerAlignment method which updates QualityReport parameter for each the SangerRead instance inside SangerAlignment.

Usage

```
## S4 method for signature 'SangerAlignment'
updateQualityParam(object,
  TrimmingMethod = "M1", M1TrimmingCutoff = 1e-04,
  M2CutoffQualityScore = NULL, M2SlidingWindowSize = NULL,
  processorsNum = NULL)
```

Arguments

`object`	A SangerAlignment S4 instance.
`TrimmingMethod`	The read trimming method for this SangerRead. The value must be `"M1"` (the default) or `'M2'`.
`M1TrimmingCutoff`	The trimming cutoff for the Method 1. If `TrimmingMethod` is `"M1"`, then the default value is `0.0001`. Otherwise, the value must be `NULL`.
`M2CutoffQualityScore`	The trimming cutoff quality score for the Method 2. If `TrimmingMethod` is `'M2'`, then the default value is `20`. Otherwise, the value must be `NULL`. It works with `M2SlidingWindowSize`.
`M2SlidingWindowSize`	The trimming sliding window size for the Method 2. If `TrimmingMethod` is `'M2'`, then the default value is `10`. Otherwise, the value must be `NULL`. It works with `M2CutoffQualityScore`.
`processorsNum`	The number of processors to use, or NULL (the default) for all available processors.

Value

A SangerAlignment instance.

Examples

```
data("sangerAlignmentData")
## Not run:
updateQualityParam(sangerAlignmentData,
                   TrimmingMethod         = "M2",
                   M1TrimmingCutoff       = NULL,
                   M2CutoffQualityScore   = 40,
                   M2SlidingWindowSize    = 15)
## End(Not run)
```

SangerAlignment-class-writeFastaSA
writeFastaSA

Description

A SangerAlignment method which writes sequences into Fasta files.

Usage

```
## S4 method for signature 'SangerAlignment'
writeFastaSA(object, outputDir = NULL,
  compress = FALSE, compression_level = NA, selection = "all")
```

Arguments

`object`	A SangerAlignment S4 instance.
`outputDir`	The output directory of generated FASTA files.
`compress`	Like for the `save` function in base R, must be `TRUE` or `FALSE` (the default), or a single string specifying whether writing to the file is to use compression. The only type of compression supported at the moment is "gzip". This parameter will be passed to `writeXStringSet` function in Biostrings package.
`compression_level`	This parameter will be passed to `writeXStringSet` function in Biostrings package.
`selection`	This value can be `all`, `contigs_alignment`, `contigs_unalignment` or `all_reads`. It generates reads and contigs FASTA files.

Value

The output directory of FASTA files.

Examples

```
data("sangerAlignmentData")
writeFastaSA(sangerAlignmentData)
```

`sangerAlignmentData` *SangerAlignment instance*

Description

SangerAlignment instance

Usage

```
data(sangerAlignmentData)
```

Author(s)

Kuan-Hao Chao

`sangeranalyseR` *sangeranalyseR-package*

Description

sangeranalyseR-package

SangerContig *SangerContig*

Description

the wrapper function for SangerContig

Usage

```
SangerContig(inputSource = "ABIF", fastaFileName = "",
  namesConversionCSV = NULL, parentDirectory = "", contigName = "",
  suffixForwardRegExp = "_F.ab1", suffixReverseRegExp = "_R.ab1",
  TrimmingMethod = "M1", M1TrimmingCutoff = 1e-04,
  M2CutoffQualityScore = NULL, M2SlidingWindowSize = NULL,
  baseNumPerRow = 100, heightPerRow = 200, signalRatioCutoff = 0.33,
  showTrimmed = TRUE, refAminoAcidSeq = "", minReadsNum = 2,
  minReadLength = 20, minFractionCall = 0.5, maxFractionLost = 0.5,
  geneticCode = GENETIC_CODE, acceptStopCodons = TRUE,
  readingFrame = 1, processorsNum = NULL)
```

Arguments

`inputSource` The input source of the raw file. It must be `"ABIF"` or `"FASTA"`. The default value is `"ABIF"`.

`fastaFileName` If `inputSource` is `"FASTA"`, then this value has to be the name of the FASTA file; if `inputSource` is `"ABIF"`, then this value is `""` by default.

`namesConversionCSV` The file path to the CSV file that provides read names that follow the naming regulation. If `inputSource` is `"FASTA"`, then users need to prepare the csv file or make sure the original names inside FASTA file are valid; if `inputSource` is `"ABIF"`, then this value is `NULL` by default.

`parentDirectory` The parent directory of all of the reads contained in ABIF format you wish to analyse. In SangerContig, all reads must be in the first layer in this directory.

`contigName` The contig name of all the reads in `parentDirectory`.

`suffixForwardRegExp` The suffix of the filenames for forward reads in regular expression, i.e. reads that do not need to be reverse-complemented. For forward reads, it should be `"_F.ab1"`.

`suffixReverseRegExp` The suffix of the filenames for reverse reads in regular expression, i.e. reads that need to be reverse-complemented. For revcerse reads, it should be `"_R.ab1"`.

`TrimmingMethod` TrimmingMethod The read trimming method for this SangerRead. The value must be `"M1"` (the default) or `'M2'`.

`M1TrimmingCutoff` The trimming cutoff for the Method 1. If `TrimmingMethod` is `"M1"`, then the default value is `0.0001`. Otherwise, the value must be `NULL`.

`M2CutoffQualityScore`
: The trimming cutoff quality score for the Method 2. If `TrimmingMethod` is `'M2'`, then the default value is `20`. Otherwise, the value must be `NULL`. It works with `M2SlidingWindowSize`.

`M2SlidingWindowSize`
: The trimming sliding window size for the Method 2. If `TrimmingMethod` is `'M2'`, then the default value is `10`. Otherwise, the value must be `NULL`. It works with `M2CutoffQualityScore`.

`baseNumPerRow` It defines maximum base pairs in each row. The default value is `100`.

`heightPerRow` It defines the height of each row in chromatogram. The default value is `200`.

`signalRatioCutoff`
: The ratio of the height of a secondary peak to a primary peak. Secondary peaks higher than this ratio are annotated. Those below the ratio are excluded. The default value is `0.33`.

`showTrimmed` The logical value storing whether to show trimmed base pairs in chromatogram. The default value is `TRUE`.

`refAminoAcidSeq`
: An amino acid reference sequence supplied as a string or an AAString object. If your sequences are protein-coding DNA seuqences, and you want to have frameshifts automatically detected and corrected, supply a reference amino acid sequence via this argument. If this argument is supplied, the sequences are then kept in frame for the alignment step. Fwd sequences are assumed to come from the sense (i.e. coding, or "+") strand. The default value is `""`.

`minReadsNum` The minimum number of reads required to make a consensus sequence, must be 2 or more. The default value is `2`.

`minReadLength` Reads shorter than this will not be included in the readset. The default `20` means that all reads with length of 20 or more will be included. Note that this is the length of a read after it has been trimmed.

`minFractionCall`
: Minimum fraction of the sequences required to call a consensus sequence for SangerContig at any given position (see the ConsensusSequence() function from DECIPHER for more information). Defaults to 0.75 implying that 3/4 of all reads must be present in order to call a consensus.

`maxFractionLost`
: Numeric giving the maximum fraction of sequence information that can be lost in the consensus sequence for SangerContig (see the ConsensusSequence() function from DECIPHER for more information). Defaults to 0.5, implying that each consensus base can ignore at most 50 percent of the information at a given position.

`geneticCode` Named character vector in the same format as `GENETIC_CODE` (the default), which represents the standard genetic code. This is the code with which the function will attempt to translate your DNA sequences. You can get an appropriate vector with the getGeneticCode() function. The default is the standard code.

`acceptStopCodons`
: The logical value `TRUE` or `FALSE`. `TRUE` (the defualt): keep all reads, regardless of whether they have stop codons; `FALSE`: reject reads with stop codons. If `FALSE` is selected, then the number of stop codons is calculated after attempting to correct frameshift mutations (if applicable).

readingFrame
: 1, 2, or 3. Only used if `accept.stop.codons == FALSE`. This specifies the reading frame that is used to determine stop codons. If you use a `refAminoAcidSeq`, then the frame should always be 1, since all reads will be shifted to frame 1 during frameshift correction. Otherwise, you should select the appropriate reading frame.

processorsNum
: The number of processors to use, or NULL (the default) for all available processors.

Value

A SangerContig instance.

Author(s)

Kuan-Hao Chao

Examples

```
rawDataDir <- system.file("extdata", package = "sangeranalyseR")
parentDir <- file.path(rawDataDir, "Allolobophora_chlorotica", "ACHLO")
contigName <- "Achl_ACHLO006-09"
suffixForwardRegExp <- "_F.ab1"
suffixReverseRegExp <- "_R.ab1"
sangerContig <- SangerContig(
                     inputSource           = "ABIF",
                     parentDirectory       = parentDir,
                     contigName            = contigName,
                     suffixForwardRegExp   = suffixForwardRegExp,
                     suffixReverseRegExp   = suffixReverseRegExp,
              refAminoAcidSeq = "SRQWLFSTNHKDIGTLYFIFGAWAGMVGTSLSILIRAELGHPGALIGDDQIYNVIVTAHAFIMIFFMVMPIMIG
                     TrimmingMethod        = "M2",
                     M1TrimmingCutoff      = NULL,
                     M2CutoffQualityScore  = 20,
                     M2SlidingWindowSize   = 10,
                     baseNumPerRow         = 100,
                     heightPerRow          = 200,
                     signalRatioCutoff     = 0.33,
                     showTrimmed           = TRUE,
                     processorsNum         = 2)
```

SangerContig-class *SangerContig*

Description

An S4 class containing forward and reverse SangerRead lists and alignment, consensus read results which corresponds to a contig in Sanger sequencing.

Slots

`inputSource` The input source of the raw file. It must be `"ABIF"` or `"FASTA"`. The default value is `"ABIF"`.

fastaFileName If inputSource is "FASTA", then this value has to be the FASTA file; if inputSource is "ABIF", then this value is NULL by default.

namesConversionCSV The file path to the CSV file that provides read names that follow the naming regulation. If inputSource is "FASTA", then users need to prepare the csv file or make sure the original names inside FASTA file are valid; if inputSource is "ABIF", then this value is NULL by default.

parentDirectory If inputSource is "ABIF", then this value is the path of the parent directory storing all reads in ABIF format you wish to analyse and cannot be NULL. In SangerContig, all reads must be in the first layer in this directory. If inputSource is "FASTA", then this value is NULL by default.

contigName The contig name of all the reads in parentDirectory.

suffixForwardRegExp The suffix of the filenames for forward reads in regular expression, i.e. reads that do not need to be reverse-complemented. For forward reads, it should be "_F.ab1".

suffixReverseRegExp The suffix of the filenames for reverse reads in regular expression, i.e. reads that need to be reverse-complemented. For revcerse reads, it should be "_R.ab1".

geneticCode Named character vector in the same format as GENETIC_CODE (the default), which represents the standard genetic code. This is the code with which the function will attempt to translate your DNA sequences. You can get an appropriate vector with the getGeneticCode() function. The default is the standard code.

forwardReadList The list of SangerRead S4 instances which are all forward reads.

reverseReadList The list of SangerRead S4 instances which are all reverse reads.

trimmingMethodSC The read trimming method for all SangerRead S4 instances in SangerContig. The value must be "M1" (the default) or 'M2'. All SangerRead must have the same trimming method.

minReadsNum The minimum number of reads required to make a consensus sequence, must be 2 or more. The default value is 2.

minReadLength Reads shorter than this will not be included in the readset. The default 20 means that all reads with length of 20 or more will be included. Note that this is the length of a read after it has been trimmed.

refAminoAcidSeq An amino acid reference sequence supplied as a string or an AAString object. If your sequences are protein-coding DNA seuqences, and you want to have frameshifts automatically detected and corrected, supply a reference amino acid sequence via this argument. If this argument is supplied, the sequences are then kept in frame for the alignment step. Fwd sequences are assumed to come from the sense (i.e. coding, or "+") strand. The default value is "".

minFractionCall Minimum fraction of the sequences required to call a consensus sequence for SangerContig at any given position (see the ConsensusSequence() function from DECIPHER for more information). Defaults to 0.75 implying that 3/4 of all reads must be present in order to call a consensus.

maxFractionLost Numeric giving the maximum fraction of sequence information that can be lost in the consensus sequence for SangerContig (see the ConsensusSequence() function from DECIPHER for more information). Defaults to 0.5, implying that each consensus base can ignore at most 50 percent of the information at a given position.

acceptStopCodons The logical value TRUE or FALSE. TRUE (the defualt): keep all reads, regardless of whether they have stop codons; FALSE: reject reads with stop codons. If FALSE is selected, then the number of stop codons is calculated after attempting to correct frameshift mutations (if applicable).

`readingFrame` 1, 2, or 3. Only used if `accept.stop.codons == FALSE`. This specifies the reading frame that is used to determine stop codons. If you use a `refAminoAcidSeq`, then the frame should always be 1, since all reads will be shifted to frame 1 during frameshift correction. Otherwise, you should select the appropriate reading frame.

`contigSeq` The consensus read of all SangerRead S4 instances in DNAString object.

`alignment` The alignment of all SangerRead S4 instances with the called consensus sequence in DNAStringSet object. Users can use BrowseSeqs() to view the alignment.

`differencesDF` A data frame of the number of pairwise differences between each read and the consensus sequence, as well as the number of bases in each input read that did not contribute to the consensus sequence. It can assist in detecting incorrect reads, or reads with a lot of errors.

`distanceMatrix` A distance matrix of genetic distances (corrected with the JC model) between all of the input reads.

`dendrogram` A list storing cluster groups in a data frame and a dendrogram object depicting the distance.matrix. Users can use plot() to see the dendrogram.

`indelsDF` If users specified a reference sequence via `refAminoAcidSeq`, then this will be a data frame describing the number of indels and deletions that were made to each of the input reads in order to correct frameshift mutations.

`stopCodonsDF` If users specified a reference sequence via `refAminoAcidSeq`, then this will be a data frame describing the number of stop codons in each read.

`secondaryPeakDF` A data frame with one row for each column in the alignment that contained more than one secondary peak. The data frame has three columns: the column number of the alignment; the number of secondary peaks in that column; and the bases (with IUPAC ambiguity codes representing secondary peak calls) in that column represented as a string.

Author(s)

Kuan-Hao Chao

Examples

```
## Input From ABIF file format (Regex)
rawDataDir <- system.file("extdata", package = "sangeranalyseR")
parentDir <- file.path(rawDataDir, "Allolobophora_chlorotica", "RBNII")
contigName <- "Achl_RBNII384-13"
suffixForwardRegExp <- "_[0-9]*_F.ab1"
suffixReverseRegExp <- "_[0-9]*_R.ab1"
sangerContig <- new("SangerContig",
                     inputSource           = "ABIF",
                     parentDirectory       = parentDir,
                     contigName            = contigName,
                     suffixForwardRegExp   = suffixForwardRegExp,
                     suffixReverseRegExp   = suffixReverseRegExp,
              refAminoAcidSeq = "SRQWLFSTNHKDIGTLYFIFGAWAGMVGTSLSILIRAELGHPGALIGDDQIYNVIVTAHAFIMIFFMVMPIMIG
                     TrimmingMethod        = "M1",
                     M1TrimmingCutoff      = 0.0001,
                     M2CutoffQualityScore  = NULL,
                     M2SlidingWindowSize   = NULL,
                     baseNumPerRow         = 100,
                     heightPerRow          = 200,
                     signalRatioCutoff     = 0.33,
                     showTrimmed           = TRUE,
```

```
                     processorsNum         = 2)

## Input From ABIF file format (Csv three column method)
rawDataDir <- system.file("extdata", package = "sangeranalyseR")
parentDir <- file.path(rawDataDir, "Allolobophora_chlorotica", "RBNII")
namesConversionCSV <- file.path(rawDataDir, "ab1", "SangerContig", "names_conversion_2.csv")
sangerContig <- new("SangerContig",
                     inputSource           = "ABIF",
                     parentDirectory       = parentDir,
                     namesConversionCSV    = namesConversionCSV,
              refAminoAcidSeq = "SRQWLFSTNHKDIGTLYFIFGAWAGMVGTSLSILIRAELGHPGALIGDDQIYNVIVTAHAFIMIFFMVMPIMIG
                     TrimmingMethod        = "M1",
                     M1TrimmingCutoff      = 0.0001,
                     M2CutoffQualityScore  = NULL,
                     M2SlidingWindowSize   = NULL,
                     baseNumPerRow         = 100,
                     heightPerRow          = 200,
                     signalRatioCutoff     = 0.33,
                     showTrimmed           = TRUE,
                     processorsNum         = 2)


## Input From FASTA file format (No Csv - Regex)
rawDataDir <- system.file("extdata", package = "sangeranalyseR")
fastaFN <- file.path(rawDataDir, "fasta",
                     "SangerContig", "Achl_ACHLO006-09.fa")
contigName <- "Achl_ACHLO006-09"
suffixForwardRegExpFa <- "_[0-9]*_F$"
suffixReverseRegExpFa <- "_[0-9]*_R$"
sangerContigFa <- new("SangerContig",
                      inputSource           = "FASTA",
                      fastaFileName         = fastaFN,
                      contigName            = contigName,
                      suffixForwardRegExp   = suffixForwardRegExpFa,
                      suffixReverseRegExp   = suffixReverseRegExpFa,
              refAminoAcidSeq     = "SRQWLFSTNHKDIGTLYFIFGAWAGMVGTSLSILIRAELGHPGALIGDDQIYNVIVTAHAFIMIFFMVMP
                      processorsNum         = 2)

## Input From FASTA file format (Csv - Csv three column method)
rawDataDir <- system.file("extdata", package = "sangeranalyseR")
fastaFN <- file.path(rawDataDir, "fasta",
                     "SangerContig", "Achl_ACHLO006-09.fa")
namesConversionCSV <- file.path(rawDataDir, "fasta", "SangerContig", "names_conversion_1.csv")
sangerContigFa <- new("SangerContig",
                      inputSource           = "FASTA",
                      fastaFileName         = fastaFN,
                      namesConversionCSV    = namesConversionCSV,
              refAminoAcidSeq     = "SRQWLFSTNHKDIGTLYFIFGAWAGMVGTSLSILIRAELGHPGALIGDDQIYNVIVTAHAFIMIFFMVMP
                      processorsNum         = 2)
```

SangerContig-class-generateReportSC

generateReportSC

Description

A SangerContig method which generates final reports of the SangerContig instance.

Usage

```
## S4 method for signature 'SangerContig'
generateReportSC(object, outputDir,
  includeSangerRead = TRUE, navigationAlignmentFN = NULL)
```

Arguments

- `object` A SangerContig S4 instance.
- `outputDir` The output directory of the generated HTML report.
- `includeSangerRead` The parameter that decides whether to include SangerRead level report. The value is `TRUE` or `FALSE` and the default is `TRUE`.
- `navigationAlignmentFN` The internal parameter passed to HTML report. Users should not modify this parameter on their own.

Value

The output absolute path to the SangerContig's HTML file.

Examples

```
data("sangerContigData")
## Not run:
generateReportSC(sangerContigData)
## End(Not run)
```

SangerContig-class-launchAppSC

launchAppSC

Description

A SangerContig method which launches Shiny app for SangerContig instance.

Usage

```
## S4 method for signature 'SangerContig'
launchAppSC(object, outputDir = NULL)
```

Arguments

- `object` A SangerContig S4 instance.
- `outputDir` The output directory of the saved new SangerContig S4 instance.

Value

A `shiny.appobj` object.

Examples

```
data("sangerContigData")
RShinySC <- launchAppSC(sangerContigData)
```

SangerContig-class-updateQualityParam
updateQualityParam

Description

A SangerContig method which updates QualityReport parameter for each the SangerRead instance inside SangerContig.

Usage

```
## S4 method for signature 'SangerContig'
updateQualityParam(object,
  TrimmingMethod = "M1", M1TrimmingCutoff = 1e-04,
  M2CutoffQualityScore = NULL, M2SlidingWindowSize = NULL,
  processorsNum = NULL)
```

Arguments

`object`	A SangerContig S4 instance.
`TrimmingMethod`	The read trimming method for this SangerRead. The value must be `"M1"` (the default) or `'M2'`.
`M1TrimmingCutoff`	The trimming cutoff for the Method 1. If `TrimmingMethod` is `"M1"`, then the default value is `0.0001`. Otherwise, the value must be `NULL`.
`M2CutoffQualityScore`	The trimming cutoff quality score for the Method 2. If `TrimmingMethod` is `'M2'`, then the default value is `20`. Otherwise, the value must be `NULL`. It works with `M2SlidingWindowSize`.
`M2SlidingWindowSize`	The trimming sliding window size for the Method 2. If `TrimmingMethod` is `'M2'`, then the default value is `10`. Otherwise, the value must be `NULL`. It works with `M2CutoffQualityScore`.
`processorsNum`	The number of processors to use, or NULL (the default) for all available processors.

Value

A SangerContig instance.

Examples

```
data("sangerContigData")
## Not run:
updateQualityParam(sangerContigData,
                   TrimmingMethod         = "M2",
                   M1TrimmingCutoff       = NULL,
                   M2CutoffQualityScore   = 40,
                   M2SlidingWindowSize    = 15)
## End(Not run)
```

SangerContig-class-writeFastaSC
writeFastaSC

Description

A SangerContig method which writes sequences into Fasta files.

Usage

```
## S4 method for signature 'SangerContig'
writeFastaSC(object, outputDir = NULL,
  compress = FALSE, compression_level = NA, selection = "all")
```

Arguments

object	A SangerContig S4 instance.
outputDir	The output directory of generated FASTA files.
compress	Like for the `save` function in base R, must be `TRUE` or `FALSE` (the default), or a single string specifying whether writing to the file is to use compression. The only type of compression supported at the moment is "gzip". This parameter will be passed to `writeXStringSet` function in Biostrings package.
compression_level	This parameter will be passed to `writeXStringSet` function in Biostrings package.
selection	This value can be `all`, `reads_alignment`, `reads_unalignment` or `contig`. It generates reads and the contig FASTA files.

Value

The output directory of FASTA files.

Examples

```
data("sangerContigData")
writeFastaSC(sangerContigData)
```

sangerContigData *SangerContig instance*

Description

SangerContig instance

Usage

```
data(sangerContigData)
```

Author(s)

Kuan-Hao Chao

SangerRead *SangerRead*

Description

the wrapper function for SangerRead

Usage

```
SangerRead(inputSource = "ABIF", readFeature = "", readFileName = "",
  fastaReadName = "", geneticCode = GENETIC_CODE,
  TrimmingMethod = "M1", M1TrimmingCutoff = 1e-04,
  M2CutoffQualityScore = NULL, M2SlidingWindowSize = NULL,
  baseNumPerRow = 100, heightPerRow = 200, signalRatioCutoff = 0.33,
  showTrimmed = TRUE)
```

Arguments

inputSource	The input source of the raw file. It must be "ABIF" or "FASTA". The default value is "ABIF".
readFeature	The direction of the Sanger read. The value must be "Forward Read" or "Reverse Read".
readFileName	The filename of the target ABIF file.
fastaReadName	If inputSource is "FASTA", then this value has to be the name of the read inside the FASTA file; if inputSource is "ABIF", then this value is "" by default.
geneticCode	Named character vector in the same format as GENETIC_CODE (the default), which represents the standard genetic code. This is the code with which the function will attempt to translate your DNA sequences. You can get an appropriate vector with the getGeneticCode() function. The default is the standard code.
TrimmingMethod	TrimmingMethod The read trimming method for this SangerRead. The value must be "M1" (the default) or 'M2'.

`M1TrimmingCutoff`
: The trimming cutoff for the Method 1. If `TrimmingMethod` is `"M1"`, then the default value is `0.0001`. Otherwise, the value must be `NULL`.

`M2CutoffQualityScore`
: The trimming cutoff quality score for the Method 2. If `TrimmingMethod` is `'M2'`, then the default value is `20`. Otherwise, the value must be `NULL`. It works with `M2SlidingWindowSize`.

`M2SlidingWindowSize`
: The trimming sliding window size for the Method 2. If `TrimmingMethod` is `'M2'`, then the default value is `10`. Otherwise, the value must be `NULL`. It works with `M2CutoffQualityScore`.

`baseNumPerRow` It defines maximum base pairs in each row. The default value is `100`.

`heightPerRow` It defines the height of each row in chromatogram. The default value is `200`.

`signalRatioCutoff`
: The ratio of the height of a secondary peak to a primary peak. Secondary peaks higher than this ratio are annotated. Those below the ratio are excluded. The default value is `0.33`.

`showTrimmed` The logical value storing whether to show trimmed base pairs in chromatogram. The default value is `TRUE`.

Value

A SangerRead instance.

Author(s)

Kuan-Hao Chao

Examples

```
inputFilesPath <- system.file("extdata/", package = "sangeranalyseR")
A_chloroticaFdFN <- file.path(inputFilesPath,
                              "Allolobophora_chlorotica",
                              "ACHLO",
                              "Achl_ACHLO006-09_1_F.ab1")
sangerRead <- SangerRead(
                   inputSource           = "ABIF",
                   readFeature           = "Forward Read",
                   readFileName          = A_chloroticaFdFN,
                   geneticCode           = GENETIC_CODE,
                   TrimmingMethod        = "M1",
                   M1TrimmingCutoff      = 0.0001,
                   M2CutoffQualityScore  = NULL,
                   M2SlidingWindowSize   = NULL,
                   baseNumPerRow         = 100,
                   heightPerRow          = 200,
                   signalRatioCutoff     = 0.33,
                   showTrimmed           = TRUE)
```

SangerRead-class *SangerRead*

Description

An S4 class extending sangerseq S4 class which corresponds to a single ABIF file in Sanger sequencing.

Slots

- `inputSource` The input source of the raw file. It must be `"ABIF"` or `"FASTA"`. The default value is `"ABIF"`.
- `readFeature` The direction of the Sanger read. The value must be `"Forward Read"` or `"Reverse Read"`.
- `readFileName` The filename of the target input file. It can be `"ABIF"` or `"FASTA"` file.
- `fastaReadName` If `inputSource` is `"FASTA"`, then this value has to be the name of the read inside the FASTA file; if `inputSource` is `"ABIF"`, then this value is `NULL` by default.
- `abifRawData` A S4 class containing all fields in the ABIF file. It is defined in sangerseqR package.
- `QualityReport` A S4 class containing quality trimming related inputs and trimming results.
- `ChromatogramParam` A S4 class containing chromatogram inputs.
- `primaryAASeqS1` A polypeptide translated from primary DNA sequence starting from the first nucleic acid.
- `primaryAASeqS2` A polypeptide translated from primary DNA sequence starting from the second nucleic acid.
- `primaryAASeqS3` A polypeptide translated from primary DNA sequence starting from the third nucleic acid.
- `geneticCode` Named character vector in the same format as `GENETIC_CODE` (the default), which represents the standard genetic code. This is the code with which the function will attempt to translate your DNA sequences. You can get an appropriate vector with the getGeneticCode() function. The default is the standard code.
- `primarySeqRaw` The raw primary sequence from sangerseq class in sangerseqR package before base calling.
- `secondarySeqRaw` The raw secondary sequence from sangerseq class in sangerseqR package before base calling.
- `peakPosMatrixRaw` The raw peak position matrix from sangerseq class in sangerseqR package before base calling.
- `peakAmpMatrixRaw` The raw peak amplitude matrix from sangerseq class in sangerseqR package before base calling.

Author(s)

Kuan-Hao Chao

Examples

```
## Input From ABIF file format
# Forward Read
inputFilesPath <- system.file("extdata/", package = "sangeranalyseR")
A_chloroticaFFN <- file.path(inputFilesPath,
                             "Allolobophora_chlorotica",
                             "ACHLO",
                             "Achl_ACHLO006-09_1_F.ab1")
sangerReadF <- new("SangerRead",
                    inputSource           = "ABIF",
                    readFeature           = "Forward Read",
                    readFileName          = A_chloroticaFFN,
                    geneticCode           = GENETIC_CODE,
                    TrimmingMethod        = "M1",
                    M1TrimmingCutoff      = 0.0001,
                    M2CutoffQualityScore  = NULL,
                    M2SlidingWindowSize   = NULL,
                    baseNumPerRow         = 100,
                    heightPerRow          = 200,
                    signalRatioCutoff     = 0.33,
                    showTrimmed           = TRUE)

# Reverse Read
A_chloroticaRFN <- file.path(inputFilesPath,
                             "Allolobophora_chlorotica",
                             "ACHLO",
                             "Achl_ACHLO006-09_2_R.ab1")
sangerReadR <- new("SangerRead",
                    inputSource           = "ABIF",
                    readFeature           = "Reverse Read",
                    readFileName          = A_chloroticaRFN,
                    geneticCode           = GENETIC_CODE,
                    TrimmingMethod        = "M1",
                    M1TrimmingCutoff      = 0.0001,
                    M2CutoffQualityScore  = NULL,
                    M2SlidingWindowSize   = NULL,
                    baseNumPerRow         = 100,
                    heightPerRow          = 200,
                    signalRatioCutoff     = 0.33,
                    showTrimmed           = TRUE)


## Input From FASTA file format
# Forward Read
inputFilesPath <- system.file("extdata/", package = "sangeranalyseR")
A_chloroticaFFNfa <- file.path(inputFilesPath,
                               "fasta",
                               "SangerRead",
                               "Achl_ACHLO006-09_1_F.fa")
readNameFfa <- "Achl_ACHLO006-09_1_F"
sangerReadFfa <- new("SangerRead",
                     inputSource        = "FASTA",
                     readFeature        = "Forward Read",
                     readFileName       = A_chloroticaFFNfa,
                     fastaReadName      = readNameFfa,
                     geneticCode        = GENETIC_CODE)
```

```
# Reverse Read
A_chloroticaRFNfa <- file.path(inputFilesPath,
                               "fasta",
                               "SangerRead",
                               "Achl_ACHLO006-09_2_R.fa")
readNameRfa <- "Achl_ACHLO006-09_2_R"
sangerReadRfa <- new("SangerRead",
                     inputSource   = "FASTA",
                     readFeature   = "Reverse Read",
                     readFileName  = A_chloroticaRFNfa,
                     fastaReadName = readNameRfa,
                     geneticCode   = GENETIC_CODE)
```

SangerRead-class-generateReportSR
generateReportSR

Description

A SangerRead method which generates final reports of the SangerRead instance.

Usage

```
## S4 method for signature 'SangerRead'
generateReportSR(object, outputDir,
  navigationContigFN = NULL, navigationAlignmentFN = NULL)
```

Arguments

`object` A SangerRead S4 instance.

`outputDir` The output directory of the generated HTML report.

`navigationContigFN` The internal parameter passed to HTML report. Users should not modify this parameter on their own.

`navigationAlignmentFN` The internal parameter passed to HTML report. Users should not modify this parameter on their own.

Value

The output absolute path to the SangerRead's HTML file.

Examples

```
data("sangerReadFData")
## Not run:
generateReportSR(sangerReadFData)
## End(Not run)
```

SangerRead-class-MakeBaseCalls
MakeBaseCalls

Description

A SangerRead method which does base calling on SangerRead instance

Usage

```
## S4 method for signature 'SangerRead'
MakeBaseCalls(object, signalRatioCutoff = 0.33)
```

Arguments

`object`	A SangerRead S4 instance.
`signalRatioCutoff`	The ratio of the height of a secondary peak to a primary peak. Secondary peaks higher than this ratio are annotated. Those below the ratio are excluded. The default value is `0.33`.

Value

A `SangerRead` instance.

Examples

```
data("sangerReadFData")
newSangerReadFData <- MakeBaseCalls(sangerReadFData, signalRatioCutoff = 0.22)
```

SangerRead-class-qualityBasePlot
qualityBasePlot

Description

A SangerRead method which creates quality base interactive plot.

Usage

```
## S4 method for signature 'SangerRead'
qualityBasePlot(object)
```

Arguments

`object`	A SangerRead S4 instance.

Value

A quality plot.

Examples

```
data("sangerReadFData")
## Not run:
qualityBasePlot(sangerReadFData)
## End(Not run)
```

SangerRead-class-readTable *readTable*

Description

A SangerRead method which generates summary table for SangerRead instance

Usage

```
## S4 method for signature 'SangerRead'
readTable(object, indentation = 0)
```

Arguments

`object`	A SangerRead S4 instance.
`indentation`	The indentation for different level printing.

Value

None

Examples

```
data(sangerReadFData)
data(sangerContigData)
data(sangerAlignmentData)
## Not run:
readTable(sangerReadFData)
readTable(sangerContigData)
readTable(sangerAlignmentData)

## End(Not run)
```

SangerRead-class-updateQualityParam
updateQualityParam

Description

A SangerRead method which updates QualityReport parameter inside the SangerRead.

Usage

```
## S4 method for signature 'SangerRead'
updateQualityParam(object, TrimmingMethod = "M1",
  M1TrimmingCutoff = 1e-04, M2CutoffQualityScore = NULL,
  M2SlidingWindowSize = NULL)
```

Arguments

- `object` A SangerRead S4 instance.
- `TrimmingMethod` The read trimming method for this SangerRead. The value must be `"M1"` (the default) or `'M2'`.
- `M1TrimmingCutoff` The trimming cutoff for the Method 1. If `TrimmingMethod` is `"M1"`, then the default value is `0.0001`. Otherwise, the value must be `NULL`.
- `M2CutoffQualityScore` The trimming cutoff quality score for the Method 2. If `TrimmingMethod` is `'M2'`, then the default value is `20`. Otherwise, the value must be `NULL`. It works with `M2SlidingWindowSize`.
- `M2SlidingWindowSize` The trimming sliding window size for the Method 2. If `TrimmingMethod` is `'M2'`, then the default value is `10`. Otherwise, the value must be `NULL`. It works with `M2CutoffQualityScore`.

Value

A SangerRead instance.

Examples

```
data("sangerReadFData")
updateQualityParam(sangerReadFData,
                   TrimmingMethod         = "M2",
                   M1TrimmingCutoff       = NULL,
                   M2CutoffQualityScore   = 40,
                   M2SlidingWindowSize    = 15)
```

`SangerRead-class-writeFastaSR` *writeFastaSR*

Description

A SangerRead method which writes the sequence into Fasta files.

Usage

```
## S4 method for signature 'SangerRead'
writeFastaSR(object, outputDir = NULL,
  compress = FALSE, compression_level = NA)
```

Arguments

`object`	A SangerRead S4 instance.
`outputDir`	The output directory of the generated FASTA file.
`compress`	Like for the `save` function in base R, must be `TRUE` or `FALSE` (the default), or a single string specifying whether writing to the file is to use compression. The only type of compression supported at the moment is "gzip". This parameter will be passed to `writeXStringSet` function in Biostrings package.
`compression_level`	This parameter will be passed to `writeXStringSet` function in Biostrings package.

Value

The output absolute path to the FASTA file.

Examples

```
data("sangerReadFData")
writeFastaSR(sangerReadFData)
```

`sangerReadFData` *SangerRead instance*

Description

SangerRead instance

Usage

```
data(sangerReadFData)
```

Author(s)

Kuan-Hao Chao

updateQualityParam *Method updateQualityParam*

Description

Method updateQualityParam

Usage

```
updateQualityParam(object, TrimmingMethod = "M1",
  M1TrimmingCutoff = 1e-04, M2CutoffQualityScore = NULL,
  M2SlidingWindowSize = NULL, ...)
```

Arguments

`object` A QualityReport, SangerRead, SangerContig, or SangerAlignment S4 instance.

`TrimmingMethod` The read trimming method for this SangerRead. The value must be `"M1"` (the default) or `'M2'`.

`M1TrimmingCutoff` The trimming cutoff for the Method 1. If `TrimmingMethod` is `"M1"`, then the default value is `0.0001`. Otherwise, the value must be `NULL`.

`M2CutoffQualityScore` The trimming cutoff quality score for the Method 2. If `TrimmingMethod` is `'M2'`, then the default value is `20`. Otherwise, the value must be `NULL`. It works with `M2SlidingWindowSize`.

`M2SlidingWindowSize` The trimming sliding window size for the Method 2. If `TrimmingMethod` is `'M2'`, then the default value is `10`. Otherwise, the value must be `NULL`. It works with `M2CutoffQualityScore`.

`...` Further updateQualityParam-related parameters.

Value

A `QualityReport`, `SangerRead`, `SangerContig`, or `SangerAlignment` instance.

Examples

```
data(qualityReportData)
data(sangerReadFData)
data(sangerContigData)
data(sangerAlignmentData)
## Not run:
updateQualityParam(qualityReportData,
                   TrimmingMethod         = "M2",
                   M1TrimmingCutoff       = NULL,
                   M2CutoffQualityScore   = 40,
                   M2SlidingWindowSize    = 15)
updateQualityParam(sangerReadFData,
                   TrimmingMethod         = "M2",
                   M1TrimmingCutoff       = NULL,
                   M2CutoffQualityScore   = 40,
```

```
                   M2SlidingWindowSize    = 15)
updateQualityParam(sangerContigData,
                   TrimmingMethod         = "M2",
                   M1TrimmingCutoff       = NULL,
                   M2CutoffQualityScore   = 40,
                   M2SlidingWindowSize    = 15)
updateQualityParam(sangerAlignmentData,
                   TrimmingMethod         = "M2",
                   M1TrimmingCutoff       = NULL,
                   M2CutoffQualityScore   = 40,
                   M2SlidingWindowSize    = 15)
## End(Not run)
```

`writeFasta` *Method writeFasta*

Description

A method which writes FASTA files of the SangerRead, SangerContig, and SangerAlignment instance.

Usage

```
writeFasta(object, outputDir = NULL, compress = FALSE,
  compression_level = NA, selection = "all")
```

Arguments

`object` A SangerRead, SangerContig, or SangerAlignment S4 instance.

`outputDir` The output directory of generated FASTA files.

`compress` Like for the `save` function in base R, must be `TRUE` or `FALSE` (the default), or a single string specifying whether writing to the file is to use compression. The only type of compression supported at the moment is "gzip". This parameter will be passed to `writeXStringSet` function in Biostrings package.

`compression_level` This parameter will be passed to `writeXStringSet` function in Biostrings package.

`selection` This parameter will be passed to `writeFastaSC` or `writeFastaSA`.

Value

A `SangerRead`, `SangerContig`, or `SangerAlignment` object.

Author(s)

Kuan-Hao Chao

Examples

```
data(sangerReadFData)
data(sangerContigData)
data(sangerAlignmentData)
## Not run:
writeFasta(sangerReadFData)
writeFasta(sangerContigData)
writeFasta(sangerAlignmentData)
## End(Not run)
```

`writeFastaSA` *Method writeFastaSA*

Description

Method writeFastaSA

Usage

```
writeFastaSA(object, outputDir = NULL, compress = FALSE,
  compression_level = NA, selection = "all")
```

Arguments

`object`	A SangerAlignment S4 instance.
`outputDir`	The output directory of generated FASTA files.
`compress`	Like for the `save` function in base R, must be `TRUE` or `FALSE` (the default), or a single string specifying whether writing to the file is to use compression. The only type of compression supported at the moment is "gzip". This parameter will be passed to `writeXStringSet` function in Biostrings package.
`compression_level`	This parameter will be passed to `writeXStringSet` function in Biostrings package.
`selection`	This value can be `all`, `contigs_alignment`, `contigs_unalignment` or `all_reads`. It generates reads and contigs FASTA files.

Value

The output directory of FASTA files.

Examples

```
data(sangerAlignmentData)
writeFastaSA(sangerAlignmentData)
```

`writeFastaSC` *Method writeFastaSC*

Description

Method writeFastaSC

Usage

```
writeFastaSC(object, outputDir = NULL, compress = FALSE,
  compression_level = NA, selection = "all")
```

Arguments

`object`	A SangerContig S4 instance.
`outputDir`	The output directory of generated FASTA files.
`compress`	Like for the `save` function in base R, must be `TRUE` or `FALSE` (the default), or a single string specifying whether writing to the file is to use compression. The only type of compression supported at the moment is "gzip". This parameter will be passed to `writeXStringSet` function in Biostrings package.
`compression_level`	This parameter will be passed to `writeXStringSet` function in Biostrings package.
`selection`	This value can be `all`, `reads_alignment`, `reads_unalignment` or `contig`. It generates reads and the contig FASTA files.

Value

The output directory of FASTA files.

Examples

```
data(sangerContigData)
writeFastaSC(sangerContigData)
```

`writeFastaSR` *Method writeFastaSR*

Description

Method writeFastaSR

Usage

```
writeFastaSR(object, outputDir = NULL, compress = FALSE,
  compression_level = NA)
```

Arguments

`object` A SangerRead S4 instance.

`outputDir` The output directory of the generated FASTA file.

`compress` Like for the `save` function in base R, must be `TRUE` or `FALSE` (the default), or a single string specifying whether writing to the file is to use compression. The only type of compression supported at the moment is "gzip". This parameter will be passed to `writeXStringSet` function in Biostrings package.

`compression_level` This parameter will be passed to `writeXStringSet` function in Biostrings package.

Value

The output absolute path to the FASTA file.

Examples

```
data(sangerReadFData)
writeFastaSR(sangerReadFData)
```

Index

∗ **datasets**
qualityReportData, 12
sangerAlignmentData, 21
sangerContigData, 31
sangerReadFData, 39

ChromatogramParam-class, 3

generateReport, 3
generateReportSA, 4
generateReportSA,SangerAlignment-method (SangerAlignment-class-generateReportSA), 18
generateReportSC, 5
generateReportSC,SangerContig-method (SangerContig-class-generateReportSC), 27
generateReportSR, 6
generateReportSR,SangerRead-method (SangerRead-class-generateReportSR), 35

launchApp, 6
launchAppSA, 7
launchAppSA,SangerAlignment-method (SangerAlignment-class-launchAppSA), 19
launchAppSC, 8
launchAppSC,SangerContig-method (SangerContig-class-launchAppSC), 28

MakeBaseCalls, 8
MakeBaseCalls,SangerRead-method (SangerRead-class-MakeBaseCalls), 36

qualityBasePlot, 9
qualityBasePlot,QualityReport-method (QualityReport-class-qualityBasePlot), 10
qualityBasePlot,SangerRead-method (SangerRead-class-qualityBasePlot), 36
QualityReport-class, 9
QualityReport-class-qualityBasePlot, 10
QualityReport-class-updateQualityParam, 11
qualityReportData, 12

readTable, 12
readTable,SangerRead-method (SangerRead-class-readTable), 37

SangerAlignment, 13
SangerAlignment-class, 16
SangerAlignment-class-generateReportSA, 18
SangerAlignment-class-launchAppSA, 19
SangerAlignment-class-updateQualityParam, 19
SangerAlignment-class-writeFastaSA, 20
sangerAlignmentData, 21
sangeranalyseR, 21
SangerContig, 22
SangerContig-class, 24
SangerContig-class-generateReportSC, 27
SangerContig-class-launchAppSC, 28
SangerContig-class-updateQualityParam, 29
SangerContig-class-writeFastaSC, 30
sangerContigData, 31
SangerRead, 31
SangerRead-class, 33
SangerRead-class-generateReportSR, 35
SangerRead-class-MakeBaseCalls, 36
SangerRead-class-qualityBasePlot, 36
SangerRead-class-readTable, 37
SangerRead-class-updateQualityParam, 38
SangerRead-class-writeFastaSR, 39
sangerReadFData, 39

updateQualityParam, 40
updateQualityParam,QualityReport-method (QualityReport-class-updateQualityParam), 11

updateQualityParam,SangerAlignment-method
(SangerAlignment-class-updateQualityParam),
19
updateQualityParam,SangerContig-method
(SangerContig-class-updateQualityParam),
29
updateQualityParam,SangerRead-method
(SangerRead-class-updateQualityParam),
38

writeFasta, 41
writeFastaSA, 42
writeFastaSA,SangerAlignment-method
(SangerAlignment-class-writeFastaSA),
20
writeFastaSC, 43
writeFastaSC,SangerContig-method
(SangerContig-class-writeFastaSC),
30
writeFastaSR, 43
writeFastaSR,SangerRead-method
(SangerRead-class-writeFastaSR),
39
