# Supplementary material for: sangeranalyseR: Simple and Interactive Processing of Sanger Sequencing Data in R
Source: Genome Biol Evol. 2021 Feb 16;13(3):evab028. doi: 10.1093/gbe/evab028 (PMC7939931; doi:10.1093/gbe/evab028)
Supplement: evab028_Supplementary_Data [file evab028_supplementary_data.zip › sangeranalyseR_vignette.htm]

An Introduction to sangeranalyseR


Code 

- Show All Code
- Hide All Code

# An Introduction to sangeranalyseR

Author: Kuan-Hao Chao (u7022787@anu.edu.au)

#### Last update: 29 June, 2020

```
library(sangeranalyseR)
```

# 1 Introduction

sangeranalyseR is an R package for analysing Sanger sequencing reads, especially those from ABIF platform, in pure R environment. There are three levels in sangeranalyseR which are ***‘SangerRead’***, ***‘SangerContig’*** and ***‘SangerAlignment’***. Users can choose which level to start the analysis. In this documentation, we intoduce analysis workflow step by step in these three levels with examples.

# 2 **SangerRead**

***‘SangerRead’*** extends from ‘sangerseq’ class and stores ‘abif’ class in sangerseqR as well as essential information including quality trimming and chromatogram parameters. It corresponds to a single ABIF file in Sanger sequencing.

## 2.1      Create **SangerRead** Instance

First step is to create a ***‘SangerRead’*** instance. Here, we find the the abosulte file path and assign it to `A_chloroticaFdReadFN`.

```
inputFilesPath <- system.file("extdata/", package = "sangeranalyseR")

A_chloroticaFdReadFN <- file.path(inputFilesPath, 
                          "Allolobophora_chlorotica",
                          "RBNII",
                          "Achl_RBNII396-13_1_F.ab1")
```

Now we can create a **‘SangerRead’** instance by running `SangerRead` constructor function.

```
singleRead <- SangerRead(readFeature   = "Forward Read",
                         readFileName  = A_chloroticaFdReadFN)
```

```
## INFO [2020-29-06 23:25:33] Forward Read: Creating abif & sangerseq ...
## INFO [2020-29-06 23:25:33]     * Creating Forward Read raw abif ...
## INFO [2020-29-06 23:25:33]     * Creating Forward Read raw sangerseq ...
## INFO [2020-29-06 23:25:33]           * Making basecall !!
## INFO [2020-29-06 23:25:34]           * Updating slots in 'SangerRead' instance !!
## SUCCESS [2020-29-06 23:25:34]   >> 'SangerRead' S4 instance is created !!
```

## 2.2      Visualize Trimmed Read

Second step is to visualize the trimmed read. `qualityBasePlot` triggers a plot\_ly interactive plot for users to check the result of the trimmed read.

```
qualityBasePlot(singleRead)
```

## 2.3      Update Trimming Parameters

Third step is to change trimming parameters. `SangerRead` constructor function uses default trimming parameters. If users are not satisfied with the trimming result, they can run `updateQualityParam` function to change the trimming parameters inside the ***‘SangerRead’*** instance.

```
updateQualityParam(singleRead,
                   TrimmingMethod         = "M1",
                   M1TrimmingCutoff       = 0.0003,
                   M2CutoffQualityScore   = NULL,
                   M2SlidingWindowSize    = NULL)
```

## 2.4      Write FASTA file

Fourth step is to export DNA sequence to FATA file. `writeFastaSR` let users to write read in ***‘SangerRead’*** instance to file in FASTA format.

```
writeFastaSR(singleRead)
```

```
## INFO [2020-29-06 23:25:35] >>> outputDir : /tmp/Rtmp2UiWTY
## INFO [2020-29-06 23:25:35] Start writing '/tmp/RtmpEgcVmy/Rinst20301817063a/sangeranalyseR/extdata//Allolobophora_chlorotica/RBNII/Achl_RBNII396-13_1_F.ab1' to FASTA format ...
## INFO [2020-29-06 23:25:35] >> '/tmp/Rtmp2UiWTY/Achl_RBNII396-13_1_F.fa' is written
```

```
## [1] "/tmp/Rtmp2UiWTY/Achl_RBNII396-13_1_F.fa"
```

## 2.5      Generate Report

Fifth step is to create a static html report for ***‘SangerRead’*** instance by running `generateReportSR` function

```
generateReportSR(singleRead)
```


---

# 3 **SangerContig**

***‘SangerContig’*** contains two lists of ***‘SangerRead’*** which are forward and reverse read list. It also contains alignment results and consensus read. It corresponds to a contig in Sanger sequencing.

## 3.1      Create **SangerContig** Instance

First step is to prepare all reads in the same directory and define the project parameters.

- `inputFilesParentDir` is the directory storing all raw ABIF files.
- `contigName` is the name of contigs. All targets share the same contig name.
- `suffixForwardRegExp` is the regular expression for forward read suffix.
- `suffixReverseRegExp` is the regular expression for reverse read suffix.

```
rawDataDir <- system.file("extdata", package = "sangeranalyseR")
inputFilesParentDir <- file.path(rawDataDir, "Allolobophora_chlorotica", "ACHLO")
contigName <- "Achl_ACHLO006-09"
suffixForwardRegExp <- "_[0-9]*_[F].ab1"
suffixReverseRegExp <- "_[0-9]*_[R].ab1"
```

After defining parameters, users can create ***‘SangerContig’*** instance by running `SangerContig` constructor function.

```
sangerContig <- SangerContig(parentDirectory      = inputFilesParentDir,
                             contigName           = contigName,
                             suffixForwardRegExp  = suffixForwardRegExp,
                             suffixReverseRegExp  = suffixReverseRegExp)
```

```
## INFO [2020-29-06 23:25:36] ******** Contig Name: Achl_ACHLO006-09
## INFO [2020-29-06 23:25:36] **** You are using Regular Expression Method to group AB1 files!
## INFO [2020-29-06 23:25:36] Forward Read: Creating abif & sangerseq ...
## INFO [2020-29-06 23:25:36]     * Creating Forward Read raw abif ...
## INFO [2020-29-06 23:25:36]     * Creating Forward Read raw sangerseq ...
## INFO [2020-29-06 23:25:36]           * Making basecall !!
## INFO [2020-29-06 23:25:36]           * Updating slots in 'SangerRead' instance !!
## SUCCESS [2020-29-06 23:25:36]   >> 'SangerRead' S4 instance is created !!
## INFO [2020-29-06 23:25:36] Reverse Read: Creating abif & sangerseq ...
## INFO [2020-29-06 23:25:36]     * Creating Reverse Read raw abif ...
## INFO [2020-29-06 23:25:37]     * Creating Reverse Read raw sangerseq ...
## INFO [2020-29-06 23:25:37]           * Making basecall !!
## INFO [2020-29-06 23:25:37]           * Updating slots in 'SangerRead' instance !!
## SUCCESS [2020-29-06 23:25:37]   >> 'SangerRead' S4 instance is created !!
## SUCCESS [2020-29-06 23:25:40]   >> 'SangerContig' S4 instance is created !!
```

## 3.2      Launch Shiny App

Second step is to trigger ***‘SangerContig’*** Shiny app. In `SangerContig` constructor function, all forward and reverse reads in this contig share the same trimming parameter by default. It is inconvenient for users to check reads one by one through R command; therefore, we provide a local Shiny app to let users easily browse and change parameters in each read in the ***‘SangerContig’*** instance.

```
launchAppSC(sangerContig)
```

## 3.3      Write FASTA file

Third step is to export DNA sequence to FATA file. After changing trimming parameters in each read, users can run `writeFastaSC` function to write results into text file in FASTA format.

```
writeFastaSC(sangerContig)
```

## 3.4      Generate Report

Fourth step is to create a report. Users can create a static html report for the ***‘SangerContig’*** instance by running `generateReportSC` function.

```
generateReportSC(sangerContig)
```


---

# 4 **SangerAlignment**

***‘SangerAlignment’*** contains a list of ***‘SangerContig’*** and the alignment results for all contigs. It corresponds to a rebuild DNA sequence fragment in Sanger sequencing.

## 4.1      Create **SangerAlignment** Instance

First step is to prepare all reads in the same directory and define the project parameters.

- `inputFilesParentDir` is the directory storing all raw ABIF files.
- `suffixForwardRegExp` is the regular expression for forward read suffix.
- `suffixReverseRegExp` is the regular expression for reverse read suffix.

```
rawDir <- system.file("extdata", package="sangeranalyseR")
parentDir <- file.path(rawDir, "Allolobophora_chlorotica", "RBNII")
suffixForwardRegExp <- "_[F]_[0-9]*.ab1"
suffixReverseRegExp <- "_[R]_[0-9]*.ab1"
```

Users can create ***‘SangerAlignment’*** instance by running `SangerAlignment` constructor function.

```
sangerAlignment <- 
        SangerAlignment(parentDirectory = parentDir,
                        suffixForwardRegExp = suffixForwardRegExp,
                        suffixReverseRegExp = suffixReverseRegExp,)
```

## 4.2      Launch Shiny App

Second step is to run `launchAppSA` to trigger the local ***‘SangerAlignment’*** Shiny app. Users can easily browse all ***‘SangerContig’*** instance in ***‘SangerAlignment’*** and change ***‘SangerRead’*** trimming parameters in each ***‘SangerContig’*** instance.

```
launchAppSA(sangerAlignment)
```

## 4.3      Write FASTA file

Third step is to run `writeFastaSC` function to write results into text file in FASTA format.

```
writeFastaSA(sangerAlignment)
```

## 4.4      Generate Report

Fourth step is to create a report. Users can create a static html report for the ***‘SangerAlignment’*** instance by running `generateReportSA` function.

```
generateReportSA(sangerAlignment)
```


---
